# SENECA: building a fully digital neuromorphic processor, design trade-offs and challenges

**DOI:** 10.3389/fnins.2023.1187252

**Published:** 2023-06-23

**Authors:** Guangzhi Tang, Kanishkan Vadivel, Yingfu Xu, Refik Bilgic, Kevin Shidqi, Paul Detterer, Stefano Traferro, Mario Konijnenburg, Manolis Sifalakis, Gert-Jan van Schaik, Amirreza Yousefzadeh

**Affiliations:** ^1^Imec, Eindhoven, Netherlands; ^2^Imec, Leuven, Belgium

**Keywords:** event-based neuromorphic processor, spiking neural network, architectural exploration, bio-inspired processing, SENECA, AI accelerator

## Abstract

Neuromorphic processors aim to emulate the biological principles of the brain to achieve high efficiency with low power consumption. However, the lack of flexibility in most neuromorphic architecture designs results in significant performance loss and inefficient memory usage when mapping various neural network algorithms. This paper proposes SENECA, a digital neuromorphic architecture that balances the trade-offs between flexibility and efficiency using a hierarchical-controlling system. A SENECA core contains two controllers, a flexible controller (RISC-V) and an optimized controller (Loop Buffer). This flexible computational pipeline allows for deploying efficient mapping for various neural networks, on-device learning, and pre-post processing algorithms. The hierarchical-controlling system introduced in SENECA makes it one of the most efficient neuromorphic processors, along with a higher level of programmability. This paper discusses the trade-offs in digital neuromorphic processor design, explains the SENECA architecture, and provides detailed experimental results when deploying various algorithms on the SENECA platform. The experimental results show that the proposed architecture improves energy and area efficiency and illustrates the effect of various trade-offs in algorithm design. A SENECA core consumes 0.47 mm^2^ when synthesized in the GF-22 nm technology node and consumes around 2.8 pJ per synaptic operation. SENECA architecture scales up by connecting many cores with a network-on-chip. The SENECA platform and the tools used in this project are freely available for academic research upon request.

## 1. Introduction

Neuromorphic engineering's vision is to boost the efficiency of neural networks to the level of the biological brain. Our brain can process temporal information from the distributed sensors, fuse them, and generate sophisticated output activities, all in real-time. In addition, it also memorizes the results and adapts to environmental changes over time (LeDoux, 1994). These tasks are done with a small energy budget of 10–20 W (Mink et al., 1981; Quian Quiroga and Kreiman, 2010). The advance of deep learning research makes neural network algorithms perform similarly or better than biological brains in many tasks (Silver et al., 2017; Brown et al., 2020; Shankar et al., 2020). However, executing those algorithms in hardware as efficiently as the brain is extremely challenging.

Since neural network algorithms are general purpose (can be applied to a variety of problems, mainly for signal processing), they enable the opportunity to build specialized hardware called Neural Processing Units (NPUs), which are simultaneously domain-specific (circuit level) and general-purpose (algorithm level). Therefore NPUs can execute neural networks with significantly more efficiency compared to CPUs. Neuromorphic processors are special NPUs that mimic biological principles by implementing features like memory-processor co-localization, sparsity exploitation, and data-flow processing. However, due to the mismatch between available silicon technology and the biological fabric of the brain, opting for the right level of bio-mimicry is the main controversial topic in this field of research. Although all state-of-the-art neuromorphic processors claim to outperform conventional solutions in specific benchmarks (Basu et al., 2022; Chen et al., 2022), they are mostly not competitive for practical applications, where a complex set of various neural networks and sensors are used (Altan et al., 2018; Grigorescu et al., 2020; Ravindran et al., 2020). Deployment of practical applications requires an end-to-end mapping of several neural network models and learning algorithms.

Despite the general-purpose attribute of neural network algorithms on their core computations, different computation pipelines on various types of neuron models, connectivity types, and learning algorithms can result in performance drops when deployed in neuromorphic platforms with an architectural mismatch. Each model of neural network requires one or a few special computation pipelines. However, a fundamental trade-off exists between making a flexible computational pipeline and an efficient processor. Most neuromorphic processors are highly efficient in executing the core computations (e.g., integrating a spike into neurons' membranes) with a specially optimized controller that limits the flexibility of the computational pipeline (Akopyan et al., 2015; Stuijt et al., 2021; Frenkel and Indiveri, 2022). The design also restricts the effective utilization of memory hierarchy and data reuse, constraining performance, area, and power efficiency. We observed that the lack of flexibility in mapping the practical applications results in significant performance loss and inefficient memory usage in such neuromorphic processors (Molendijk et al., 2022), making them a non-competitive solution for the EdgeAI market. On the other hand, several recent neuromorphic processors opted for a high level of programmability by using a complex and less efficient controller (for example, an embedded processor) to schedule their computational pipeline flexibly (Davis, 2021; Höppner et al., 2021). Benchmarking results in this work show such a controller could consume an order of magnitude more energy than an efficient controller. Therefore, an effective neuromorphic architecture design is needed to balance the trade-off between flexibility and efficiency.

In this paper, we propose the SENECA neuromorphic architecture, a flexible and efficient design with a hierarchical controlling system consisting of a flexible controller (RISC-V) and a custom-made efficient controller (Loop Buffer). During computation, the loop buffer executes micro-codes made by a series of simple instructions, and RISC-V controls the order of execution of each micro-code, which makes the computational pipeline customizable and efficient. Moreover, the multi-level flexible controller enables SENECA to employ a hierarchical memory structure with an efficient data reuse capability. Such an architecture gives SENECA a high level of flexibility and area efficiency without sacrificing energy efficiency. We showed that SENECA is among the most energy-efficient neuromorphic processors while keeping its high level of flexibility. Briefly, the main contributions of the paper are the following:

- Introduce a neuromorphic processor with a flexible processing pipeline to efficiently deploy various neuron models, connectivity types, and learning algorithms on one platform.
- Introduce the concept of the hierarchical control mechanism that allows for high flexibility without significant performance loss.
- Provide detailed measurements of energy consumption of various logic blocks, neuron processing instructions, and neural network algorithms in SENECA, which is helpful for future design space exploration and algorithm-hardware co-optimization.
- Demonstrate spike-grouping as a method to exploit the memory hierarchy and improve the energy efficiency of neuromorphic processing.

We discuss the trade-offs in the design of a digital neuromorphic processor and compare state-of-the-art architectures based on those trade-offs in Section 2. We introduce the SENECA neuromorphic architecture in Section 3. This architecture was briefly introduced in Yousefzadeh et al. (2022). In this paper, we provide more extensive architectural details. We also explain the design choices of SENECA based on the mentioned trade-offs. Synthesis results, instruction level, and algorithm level benchmarking of the SENECA processor are provided in Section 4. The provided results can be useful for modeling in algorithm-hardware co-optimizations. Our synthesis result shows SENECA has a high area efficiency, and the instruction level benchmarking showed a competitive 2.8 pJ/Synaptic operation when employing a data reuse strategy. Algorithm level benchmarking in Section 4.2 shows SENECA's performance for fully connected and convolutional neural networks next to the on-device learning with e-prop. Algorithm-level benchmarking provides more insight into instruction-level benchmarking by measuring all the overheads of the RISC-V controller. Our experimental results showed that the flexibility overhead provided by RISC-V is bounded within 10% the main bulk of the computational load. It also demonstrates the flexibility of SENECA to map various neural network algorithms efficiently. The paper ends with a short conclusion in Section 5.

## 2. Important trade-offs in neuromorphic architecture design

Since neuromorphic architecture design aims to follow the principles of bio-inspired processing mechanisms in the available nano-electronic technologies, facing several challenges that result from the platform constraints is expected. In this section, we discuss the challenges of neuromorphic architecture design by reviewing existing approaches and justifying the choice of SENECA design with trade-offs in solving these challenges.

### 2.1. Logic time-multiplexing

A biological neural network is built from many neurons connected through synapses and axons. Neurons contain an internal state (so-called membrane potential) that accumulates weighted spikes. In its simplest form, each synapse has a weight that adjusts the intensity of the current injected into the post-synaptic neuron.

**Figure 1:**
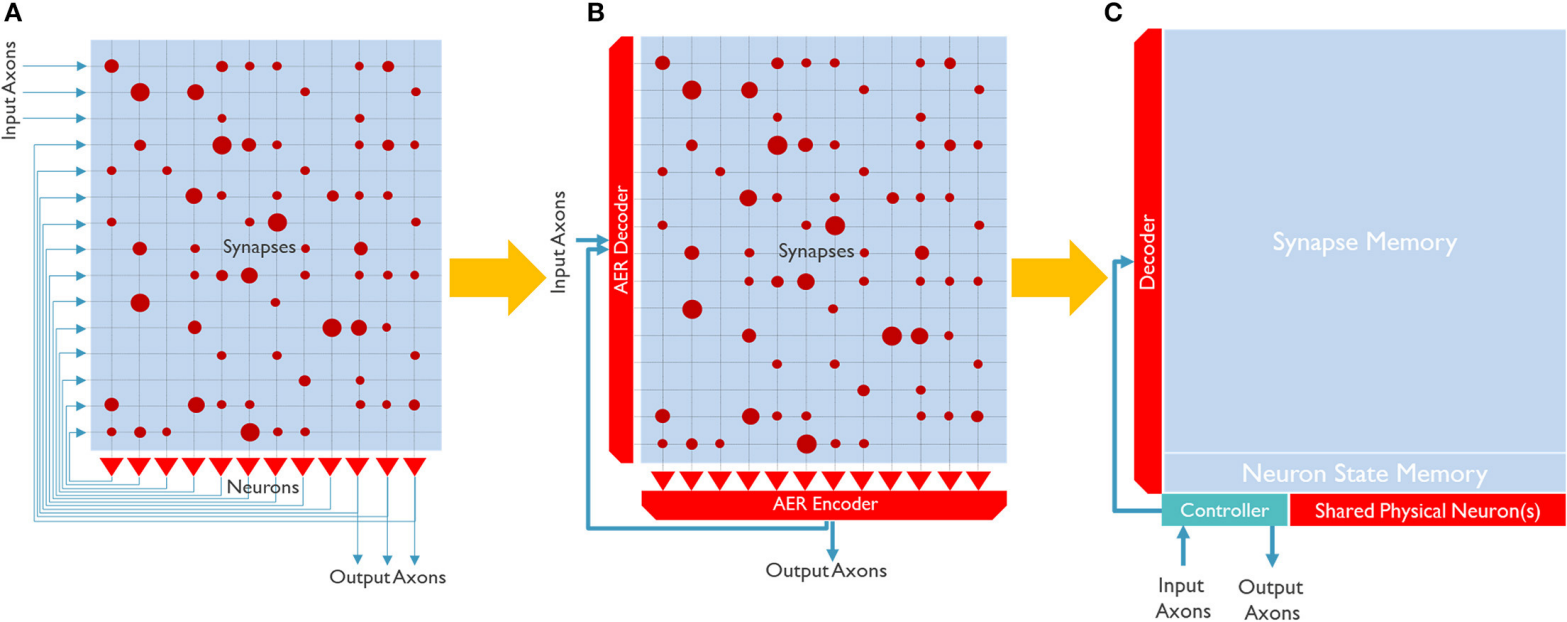
Simple block diagrams of common neuro-synaptic cores. **(A)** Shows a neuro-synaptic core that implements 12 neurons, each with 15 synapses and one output axon. The core has 3 input axons, and 3 output axons and neurons are fully connected. The red dots in the synaptic array show the synaptic weight's strength. **(B)** Shows a neuro-synaptic core where the axons are time-multiplexed and implemented with a shared connection. **(C)** Shows a neuro-synaptic core where both the axons and neurons are time-multiplexed.

Figure 1 shows three simplified examples of architectures for a neuro-synaptic core that emulates a population of interconnected neurons. These architectures can be realized in silicon with various technologies [for example, analog (Schemmel et al., 2022), digital (Arthur et al., 2012; Stuijt et al., 2021), and in-memory processing (Ahmadi-Farsani et al., 2022)]. Figure 1A is the most bio-inspired one, in which neurons are interconnected through a cross-bar synaptic memory. However, this explicit connectivity can become easily prohibitive to be routed in a 2D structure of conventional silicon ICs.

Since data can travel/process a million times faster in silicon than in the brain^1^, a typical silicon neural network can operate 1*M* times faster than its biological counterpart. Therefore, it makes sense to partially time-multiplex elements of silicon neural networks, even though it is not bio-inspired. To the best of our knowledge, all scalable digital neuromorphic chips adopt a kind of time-multiplexing technique.

**Figure 2:**
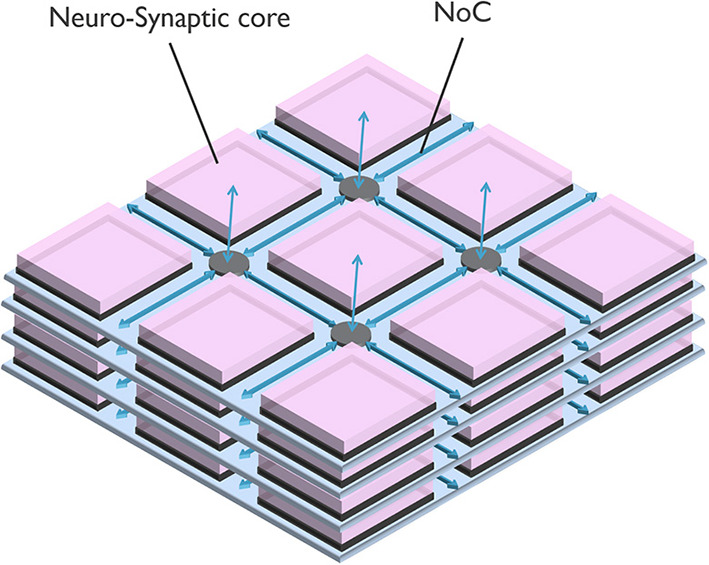
A typical neuromorphic processor, scaling with a Network on Chip (NoC) as time-multiplexed axons.

Figure 1B illustrates a neuro-synaptic core architecture where the axons are time-multiplexed. In this case, each spike pulse is encoded in a packet of data, including the address of the source neuron, so-called Address Event Representation (AER) (Yousefzadeh et al., 2017a). Using this method, each neuron needs to process one spike at a time (in series), simplifying silicon neurons' architecture. In addition, axon time-multiplexing allows flexibility and scalability by connecting many neuro-synaptic cores in a packet-switched network, as shown in Figure 2. However, axon time multiplexing changes a single spike pulse to a potentially large data packet. For example, in Figure 1B, each packet will need to have at least *log*_2_(*number of neurons*) bits to accommodate the address of neurons.

Despite the packetization overhead, axon time multiplexing is used in all digital neuromorphic processors (to our knowledge). A step further is to time-multiplex the physical neurons, as shown in Figure 1C. In this case, one shared physical neuron can emulate several hundreds of neurons in a neuro-synaptic core. Especially when the neuron model is more complex, or the physical neuron is designed to be programmable (for example, to support several neuron models), neuron time multiplexing significantly improves the area efficiency and allows to scale up the number of neurons in a neuro-synaptic core. However, it also introduces some serious trade-offs.

First, time multiplexing of neurons requires loading and storing the neuron states from memory. In Figure 1B, neuron states can remain inside the physical neurons. But in Figure 1C, a neuron state memory is introduced. Each physical neuron needs to load the corresponding neuron state, update it and store it back in the neuron state memory. This extra memory access potentially reduces the system's energy efficiency. Additionally, even though silicon is much faster than bio-fabric, in practice, neuron time-multiplexing can slow down the neuro-synaptic cores and increase latency. The controller in Figure 1C is the second overhead of neuron time-multiplexing. Neuron time-multiplexing requires a controller to orchestrate the time-multiplexing process. The complexity of the controller depends on the flexibility (programmability) and features that the core supports.

Despite the disadvantage of neuron time-multiplexing, its benefits in area efficiency make it inevitable to be used by almost all digital neuromorphic processors (Furber et al., 2014; Akopyan et al., 2015; Davies et al., 2018; Frenkel et al., 2018; Demler, 2019; Mayr et al., 2019; Moreira et al., 2020; Davis, 2021). However, for some small-scale architectures (sub *mW*) (Stuijt et al., 2021) where power consumption is prioritized over area efficiency and programmability, the controller overhead might be considerable, and therefore neuron time-multiplexing is not implemented. The last column of Table 1 shows a few neuromorphic architectures and their neuron time-multiplexing ratio. SENECA uses axon and neuron time-multiplexing to process a flexible number of neurons in each core.

**Table 1:** Comparison between area, memory, and the technology node used in a few neuromorphic chips.

**Architecture**	***mm* ^2^**	**Memory (*Mb*)**	**Technology**	**Neurons(Physical)**
	**/Core**	**/Core**		**/core**
ODIN (Frenkel et al., 2018)	0.086	0.28	STM 28 nm	256(1)
TrueNorth (Akopyan et al., 2015)	0.10	0.10	Samsung 28 nm	256(1)
NeuronFlow (Moreira et al., 2020)	0.1	0.12	TSMC 28 nm	1024(1)
Loihi2 (Davis, 2021)	0.21	1.5	Intel 4 nm	Flex(1)
Loihi (Davies et al., 2018)	0.41	2.0	Intel 14 nm	1024(1)
ReckOn (Frenkel and Indiveri, 2022)	0.45	1.1	FDSOI 28 nm	256(16)
μBrain (Stuijt et al., 2021)	1.42	0.15	TSMC 40 nm	336(336)
SpiNNaker (Furber et al., 2014)	5.6	0.12	130 nm	Flex(1)
SpiNNaker2 (Höppner et al., 2021)	1.09	1.0	FDX 22 nm	Flex (64)
Tianjic (Deng et al., 2020)	0.092	0.17	UMC 28 nm	256(16)
SENECA	0.47	2.3	FDX 22 nm	Flex(8)

The amount of memory can be used as an indication of the number of neurons and synapses per core.

### 2.2. Memory

In the architecture shown in Figure 1C, memory cells are the only part that cannot be time-multiplexed. Each neuron must have dedicated memory cells for membrane potential (neuron state), synaptic weights, and axons (destination addresses). As a result, memory is responsible for most of the area and power consumption in a neuro-synaptic core.

Several trade-offs are involved in the design of the memory block (Stansfield, 2022). The first trade-off regarding memory is the size of memory per core. As a rule of thumb, the area efficiency in a neuro-synaptic core improves by increasing the memory size (due to an increase in the time-multiplexing ratio of other elements). However, the higher time-multiplexing ratio for the physical neurons, in general, increases the processing time. Additionally, the distance between the memory cells and its peripherals increases in a larger memory, resulting in slightly higher power consumption of individual memory accesses. On the other hand, using smaller memory in the core means less number of neurons/synapses per core. Therefore, in such a platform, it is required to use more interconnected cores to deploy an application, which also increases the load of the interconnect (more data movement). Table 1 shows the amount of memory per core in a few digital neuromorphic processors.

A second challenge is the choice of memory technology. Register-File (Latch) and SRAM^2^ are the most common memories used in digital processors. New memory technologies (eDRAM, eFlash, MRAM, etc.) are also gaining popularity. It is also possible to opt for off-chip memory. In this case, the method to connect two chips to each other greatly affects the performance (2D chiplet, 3D stacked integration, etc.).

μBrain (Stuijt et al., 2021) uses latch memory, which allows it to be fully synthesizable (SRAMs are analog IPs and cannot be synthesized using standard digital gates). Register Files (and Latches) are fully synthesizable using the standard digital gates (unlike SRAM, which is an analog IP); therefore, placing each memory cell very close to the processing logic is possible. However, it consumes more area than SRAM for larger memory sizes (Teman et al., 2016). SRAM provides a very competitive balance for the area, performance and power consumption when only one memory technology is used. As a result, most of the architectures in Table 1 only use SRAM memory for weight and neuron states. However, when targeting large-scale neural networks (multi-Gb parameters), SRAM becomes unaffordable. SpiNNaker (Furber et al., 2014) uses SRAMs for neuron states and a 1Gb 3D-integrated off-chip DDR memory for synaptic weights (in its standard SDK). This arrangement allows for storing a large number of synaptic weights (1Gb) in a small, affordable chip. Using off-chip DDR memory dramatically improves the area efficiency and cost since memory foundries optimize the process of memory cells for large-scale fabrication (for example, by using fewer metal layers). However, it also increases the distance between memory and the processor, which is undesirable, especially for neuromorphic processors (Pedram et al., 2016).

Due to a highly sparse processing pattern of neuromorphic applications, the static power consumption in a neuromorphic chip, if not carefully designed, can easily exceed 30% of the total power consumption (Stuijt et al., 2021). Data retention in volatile memory is the primary source of static power consumption. Using Non-Volatile Memory (NVM) technologies can theoretically address this issue. However, NVM technologies generally suffer from high latency (access time), extremely high write power consumption and limited endurance.

SENECA architecture is designed to use a hybrid memory architecture and mixed memory technologies. SENECA has the flexibility to dynamically allocate different parameters to various memory blocks. Therefore, one can optimize the application mapping for the best energy and area trade-offs. In this case, the data's location will depend on how often it is used. Table 2 shows Power, Performance, and Area (PPA) measurements of different memory technologies used in SENECA, measured using randomized experiments in Cadence JOULES (with FDX 22 nm technology, typical corner). As can be seen, each memory technology has its own unique advantages, which can be optimized when used in a hybrid memory architecture.

**Table 2:** Comparison of memory modules used in SENECA.

**Memory module**	**Memory size**	**Energy**	**Static power**	**Area**	**Latency**
		**(*fJ*/b)**	**(*pW*/b)**	**(*um*^2^/b)**	**(*ns*)**
Register-file (inside NPEs)	16*W*×16*b* (256*b*)	8	600	3.6	<1
	64*W*×16*b* (1*kb*)	12	610	3.6	<1
SRAM block (Inst/Data Mem)	8*KW*×32*b* (256*Kb*)	180(R)–220(W)	10	0.2	2
STT-MRAM (Shared Mem)	256*k*×144*b* (36.8*Mb*)	2,000(R)	0	0.1	25(R)
HBM (Shared Mem; Xilinx, 2020)	64 Gb	7000	–	0.003	135

### 2.3. Programmability

Programmability means “The capability within hardware and software to change; to accept a new set of instructions that alter its behavior.” In this definition, the biological brain is programmable. Our brain easily adapts to the augmented artificial sensors and actuators (Hartmann et al., 2016).

The desired level of programmability in the neuromorphic processors is much higher than in the brain. At least, a user of a neuromorphic processor needs to start from a pre-trained network and be able to program the synaptic weights. In addition, there are various neural network architectures, learning algorithms, and neuron models. A highly flexible neuromorphic processor allows the deployment of several applications and algorithms and is helpful in researching and developing new ideas.

Adding flexibility to the architecture will cost area and power. Thereby increasing the energy consumption per operation. However, the added functionalities may result in optimizations that significantly improve the application level performance, for example, by reducing data movement and memory access. Table 3 lists a few neuromorphic architectures based on their level of programmability. A flexible mapping allows for reusing all memory blocks for neurons and synapses to use the maximum amount of memory in each core (no hard partitioning of memories). Using programmable data type allows for the optimal mapping of quantized networks. Different layers in a neural network have different quantization requirements, which can only be exploited if the processor supports multiple data types.

**Table 3:** Programmibility (flexibility) of different dimensions in different neuromorphic processors.

**Architecture**	**Mapping**	**Data-type**	**Network**	**Neuron**	**Synapse**	**Energy per**
			**architecture**	**model**	**model**	**SOp (*pJ*)**
ODIN	Low	Fixed	Fixed	Fixed	Fixed	12.7
(Frenkel et al., 2018)						
ReckOn	Low	Fixed	Fixed	Fixed	Fixed	5.3
(Frenkel and Indiveri, 2022)						
μBrain	Low	Fixed	Fixed	Fixed	Fixed	26
(Stuijt et al., 2021)						
TrueNorth	Low	Fixed	Low	Fixed	Fixed	2.5
(Akopyan et al., 2015)						
Tianjic	Low	Fixed	High	Medium	Fixed	1.54
(Deng et al., 2020)						
NeuronFlow	Low	Low	Medium	Medium	Fixed	20
(Moreira et al., 2020)						
Loihi	Low	Low	Medium	Low	Medium	23.6
(Davies et al., 2018)						
Loihi2	High	Medium	Medium	High	Medium	NA
(Davis, 2021)						
SpiNNaker	High	Medium	High	High	High	45
(Stromatias et al., 2013)						
SpiNNaker2	High	Medium	High	High	High	10
(Höppner et al., 2021)						
SENECA	High	Medium	High	High	High	2.8

Synaptic Operation (SOp) varies in different applications and is only mentioned for high-level comparison. **Mapping**: low—hard partitioning of memory for weight and state; high—flexible memory reusing. **Data-type**: fixed—single data type supported; low—limited data type supported and only support binary events; medium—mixed-precision data type supported and graded events supported. **Network architecture**: fixed—only support Fully-Connected network; low—optimal support on Fully-Connected network and very costly support on CNN; medium—optimal support on Fully-Connected network and costly support to CNN; high—optimal support to both fully-Connected and CNN, and can also support novel network architectures. **Neuron model**: fixed—single fixed model; low—single predefined model with limited programmability; medium—multiple predefined models with limited programmability; high—fully programmable model. **Synapse model**: fixed—single fixed model; medium—single fixed model with limited programmable learning support; high—fully programmable model and fully programmable learning support.

Supporting efficient deployment of various neural network architectures (Dense, Conv, RNN, Transformers, etc.) also requires flexibility. For example, in most neural networks, due to a regular architecture, it is possible to mathematically calculate the destination address of a neuron (in the controller in Figure 1C) instead of storing them in the axon memory, therefore saving a relatively large amount of memory. Another example is weight sharing in Convolutional Neural Networks (CNNs). In CNNs, synapses of a channel share their weights. If the processor architecture cannot support the weight-sharing feature, it is required to store several hundred copies of the same synaptic weights in the weight memory. For example, Implementation of an HW-optimized CNN in TrueNorth (Akopyan et al., 2015) with 1.5 M ternary weights (3 Mb), consumed 3, 721 cores (372 Mb; Amir et al., 2017). Mapping of the same CNN in SENECA requires only 4 cores (8.4 Mb).

Supporting various neuron and synaptic models (e.g., plasticity algorithms) requires additional flexibility. At this moment, there is no evidence that a specific spiking neuron model or a local learning algorithm will be dominant due to its superior efficiency in all applications. Therefore, these flexibilities can result in better power consumption when considering end-to-end application deployment. In particular, the local learning algorithm within the synapse model, which is at the frontier of neural network research, requires the right level of programmability to explore application-level performance optimizations. For example, Davies et al. (2018) provides configurable learning rules using microcode operations supported by the learning engine per core. By limiting flexibility to the sum-of-products of synaptic traces, Loihi struggled to deploy advanced learning algorithms and required algorithm designers to find non-optimal workarounds to deploy the learning on the chip (Renner et al., 2021; Tang et al., 2021). Furthermore, trace-based learning on Loihi requires updating all synapses at each time step, restricting the learning algorithm from exploiting the event-driven advantage of neuromorphic computing^3^. In contrast, SENECA is at the right level of programmability to deploy various learning algorithms via the neuron processing instruction set (detailed in Section 3.2), which can better exploit the application-level performance optimization.

### 2.4. Interconnectivity

To connect the neuro-synaptic cores to each other in a neuromorphic system (Figure 2), it is possible to use shared buses (or circuit-switched Network on Chip [NoC]; Balaji et al., 2019), point-to-point connections (Stuijt et al., 2021), or a packet-switched NoC. The packet-switched NoC is the most popular option due to its higher performance and flexibility (Furber et al., 2014; Akopyan et al., 2015; Moradi et al., 2017; Davies et al., 2018; Frenkel et al., 2018; Demler, 2019; Mayr et al., 2019; Moreira et al., 2020; Davis, 2021), as shown in Table 4.

**Table 4:** Type of network on chip for different large-scale neuromorphic processors.

**Architecture**	**Core/Router**	**Multicasting**	**Compression**
TrueNorth (Akopyan et al., 2015)	1	No	No
NeuronFlow (Moreira et al., 2020)	1	No	No
Loihi (Davies et al., 2018)	4	No	No
Loihi2 (Davis, 2021)	4	No	No
SpiNNaker (Stromatias et al., 2013)	16	Yes	No
SpiNNaker2 (Mayr et al., 2019)	4	Yes	Software
Tianjic (Deng et al., 2020)	1	Yes	No
SENECA	1	Yes	Software

One of the challenges in neuromorphic chips is the “operational intensity” of a single packet of data. In other words, if the processing of a packet of data is much faster than the delivery time of that packet (low operational intensity), then the interconnect is the main bottleneck. For example, a spike from an axon that is connected to many neurons triggers a high amount of neural updates. If all destination neurons are located in one neuro-synaptic core, then the operational intensity of the spike packet is high. However, if the destination neurons are distributed in several cores, many spike packets are required to deliver the same spikes to all of those cores. In this case, the operational intensity of each packet is lower. Therefore, in the platforms with smaller but more cores, the spike packets' operation density is generally lower.

Multi-casting is a feature that increases the operational intensity of spike packets by reducing the total data movements. When a core wants to send a spike packet to several other cores in a uni-cast interconnect, several copies of the packet with different destination addresses must travel over the interconnect from the source core toward the destination cores. A multicasting NoC makes the copies closer to the destination cores, reducing the communication overhead. As a trade-off, complete support of multi-casting considerably increases the complexity of the NoC. SENECA supports a lightweight multi-casting NoC with a small routing table. Our study showed that a small routing table is enough for most of the neural networks with regular connectivity. However, SENECA NoC needs to switch to the unicast mode in extreme irregularity cases.

Another possibility to improve the operation intensity is to compress the spike packets. Each spike packet contains an address field and an optional data field. It is easier to compress the address field for the spikes which are fired simultaneously since they are generally correlated. Spike compression saves the NoC energy and the memory consumption of spike queues (at the entrance of each core). However, a compression algorithm introduces extra computational overhead, and its performance is application dependent. Therefore, selecting a compression algorithm and accelerating it in a neuromorphic processor is a difficult trade-off. Additionally, due to spike compression, the spike packets will have variable lengths, which slightly increases the router's complexity. In SENECA, we use a simplified yet effective spike address compression inside the controller.

### 2.5. Asynchronous design

Another challenge for digital event-based processing cores is the clock. At this moment, synchronous digital design, which requires a clock signal, is far more popular than asynchronous design. The main reason is that, in synchronous digital design, the circuit's behavior is not dependent on the timing characteristics of the underlying silicon technology.

**Figure 3:**
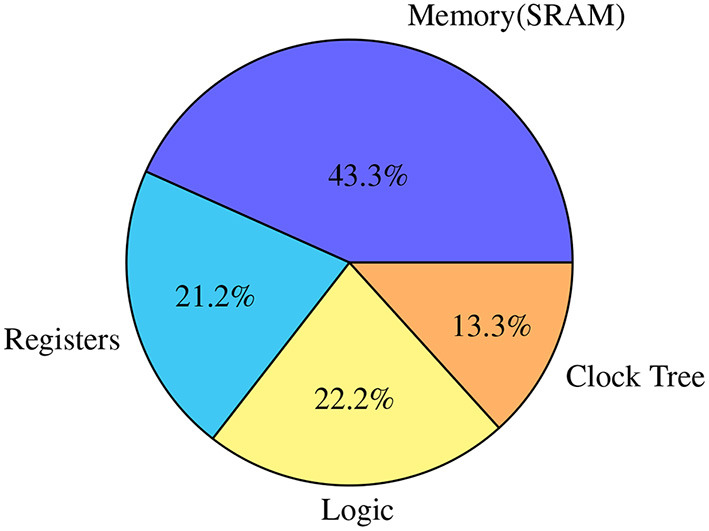
Distribution of the power consumption in various elements in SENECA when executing the online learning algorithm experiment, explained in Section 4.2.

A clock is a high-frequency pulse that is continuously switching. In a synchronous digital circuit, the clock signal must reach almost everywhere inside a synchronous domain through a highly controlled latency circuit (called a clock tree). As shown in Figure 3, a clock tree consumes a substantial part of the dynamic energy in SENECA, which is a significant overhead.

A traditional method to address the wasted dynamic power of the system due to clock signal in idle time is clock gating. In this method, a control logic gates the clock signal of a digital block in the absence of events. However, due to the overhead of the control logic, it is not feasible to reach 100% clock gating efficiency.

One possibility to improve the system's scalability is to have regional clock generators. In this case, a large clock tree is divided into smaller local trees. This method is called GALS (Globally asynchronous, Locally Synchronous) architecture. In GALS, the interconnects between these synchronous regions must follow an asynchronous protocol (Yousefzadeh et al., 2016). The trade-off in GALS design is drawing the asynchronous boundary, which can be either inside their cores, between cores, or between chips for large-scale designs.

A possible optimization over GALS is designing clock generators triggered by the input events. In this so-called **self-clocked logic**, a distributed set of simplified oscillator circuits generates the exact number of pulses required to process an input event. Therefore, clock gating latches are not required (which are constantly active). Depending on the number of event-based oscillators, a self-clocked circuit design might be a better trade-off than clock gating. However, it requires a complex, hand-optimized asynchronous digital design, which affects its design cost and portability to newer technologies.

Asynchronous design is advertised to be faster and consumes less dynamic power. However, without spending considerable design time, it isn't easy to harvest its benefits. On the other hand, synchronous circuits can be designed to finish their task extremely fast and turn off the clock signal to save energy. Considering those trade-offs, we only used GALS with core-to-core asynchrony in the SENECA. Table 5 lists several neuromorphic processors based on their asynchronous design choices.

**Table 5:** Asynchronousity level in various neuromorphic chips.

**Architecture**	**Asynchronousity level**
ODIN (Frenkel et al., 2018)	Fully synchronous
ReckOn (Frenkel and Indiveri, 2022)	Fully synchronous
μBrain (Stuijt et al., 2021)	Self-clocked
TrueNorth (Akopyan et al., 2015)	Core-to-core
NeuronFlow (Moreira et al., 2020)	Chip-to-chip
Loihi (Davies et al., 2018)	Self-clocked
Loihi2 (Davis, 2021)	Self-clocked
SpiNNaker (Stromatias et al., 2013)	Core-to-core
SpiNNaker2 (Mayr et al., 2019)	Core-to-core
Tianjic (Deng et al., 2020)	Core-to-core
SENECA	Core-to-core

## 3. SENECA architecture

**Figure 4:**
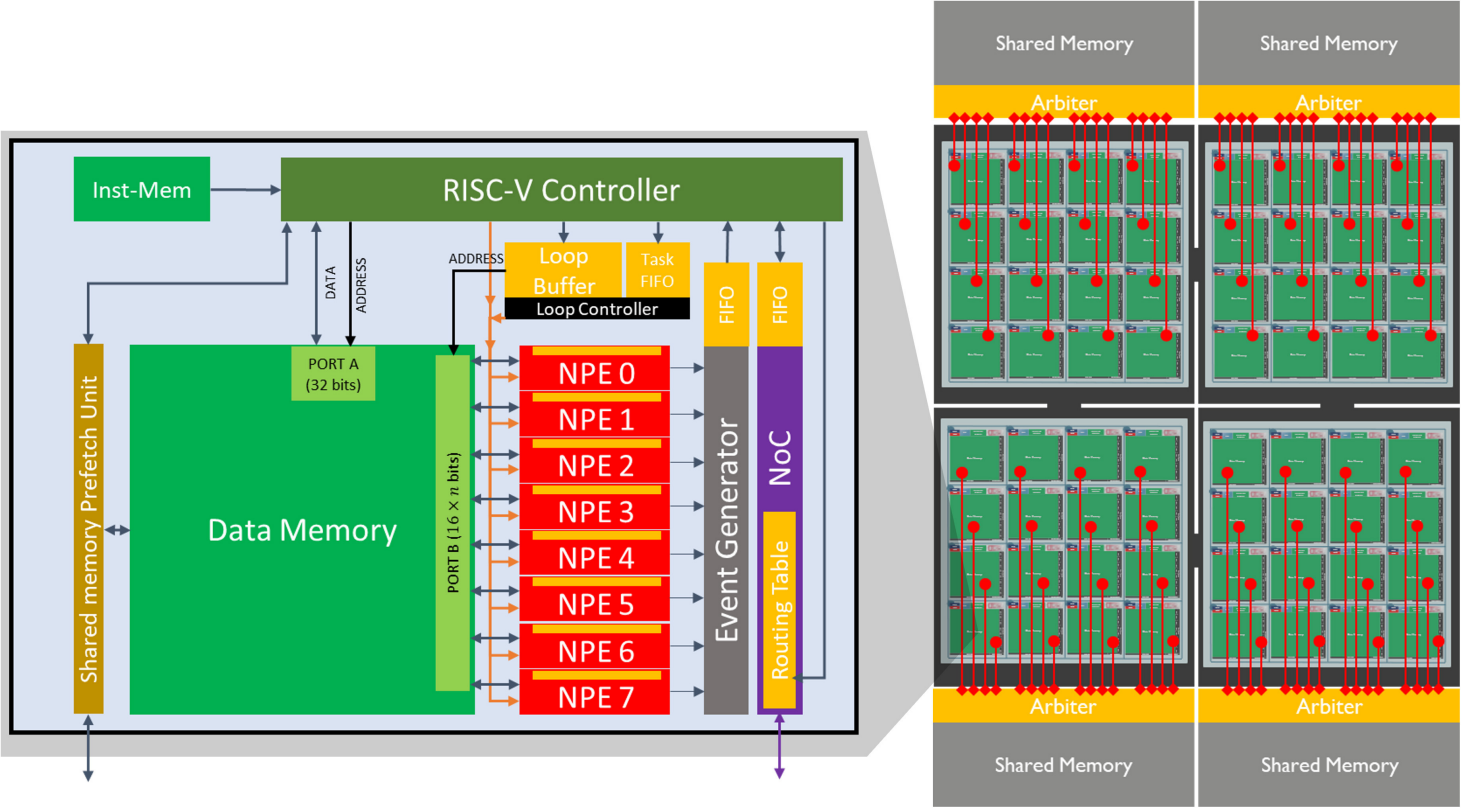
**(Left)** A core of SENECA and its internal pipeline. It contains a general-purpose controller (RISC-V), many Neuron Processing Elements (NPEs) as physical neurons, Loop Buffer, Event-generator, NoC, and Share Memory Prefetch Unit. The orange blocks are the register-based memories, and the green blocks are the SRAM memories. **(Right)** Four interconnected clusters containing 16 SENECA cores (connected through the NoC) and one shared memory (MRAM or HBM).

In this section, we introduce our proposed neuromorphic architecture, named SENECA^4^. SENECA comprises interconnected neurosynaptic cores, illustrated in Figure 4. The cores are programmed to process input events and generate output events. An input event enters the core through the NoC (Network on Chip) and interrupts the RISC-V. Depending on the type of event, RISC-V decides how to preprocess it. In general, for a normal incoming spike, RISC-V performs a pre-processing phase to retrieve the relevant local information required to process the spikes (for example, the address of the corresponding parameters in the Data Memory) and packs that information in the form of a micro-task. Then this micro-task is pushed to the Task FIFO. The loop controller executes the tasks one by one based on the micro-code instructions stored in the loop buffer. The loop controller is a small dedicated controller programmed to execute a sequence of instructions in parallel through the NPEs (Neural Processing Elements). Some neural operations in NPEs may result in output spikes which will be converted to packets of data inside the event generator. The event generator unit interrupts the RISC-V to perform postprocessing on the generated events. RISC-V can feed the generated events back into the Task FIFO or send them out through the NoC. Following, we will explain each element of SENECA in more detail.

### 3.1. RISC-V controller

In Figure 1C, there is a controller which handles the input/output spike flow. This controller mainly performs the address translation task. It generates an address for the newborn spikes from the physical neuron and translates the addresses of the incoming spikes to the internal memory address. Address translation depends on the architecture and mapping of the neural network. A general-purpose processor allows for efficient mapping of various applications, improving both area and power efficiency.

In SENECA, we used a tiny RISC-V as part of the controller of the core. This controller (along with its instruction memory) consumes around 10% of the total core area, and its energy efficiency is around 10 × worse than the accelerated neural processing element (NPE). However, if properly used, it provides features that well-compensates the costs through:

1. Dynamic allocation and reuse of the core memory for both weights and neuron states.
2. Calculate the destination address of neurons (axons) instead of using axon memory.
3. The optimum use of different memory technologies.
4. Implementing a lightweight event-compression mechanism.

**Figure 5:**
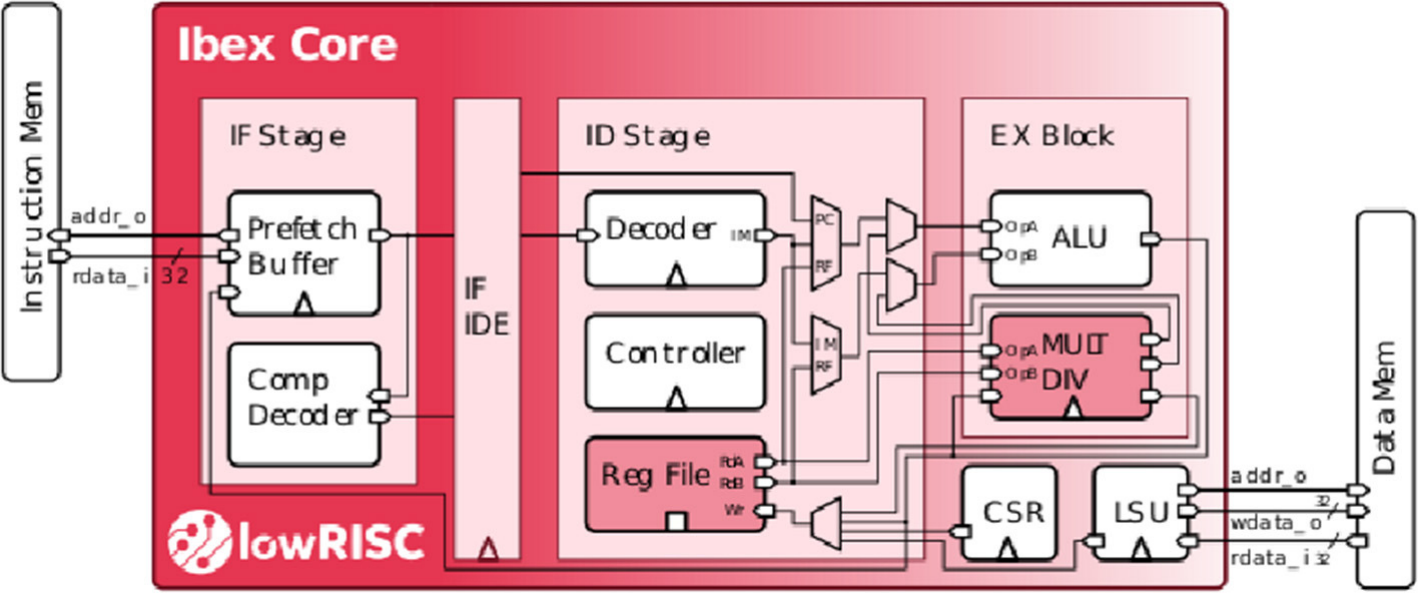
Internal structure of the Ibex controller (Schiavone et al., 2017; Chadwick, 2018).

RISC-V performs per-spike operations (not per synapse). For many popular neural network architectures, each spike(activation) triggers over 100 synaptic updates (Yousefzadeh and Sifalakis, 2022). As part of the address calculation is accelerated in the Event Generator (output spikes) and in the Loop controller (input spikes), RISC-V only executes <1% of the total number of operations in a target application. This results in a negligible energy overhead which can be compensated by optimized memory access. The selected RISC-V controller in a SENECA core is a low-power, free and open-source Ibex controller from lowRISC^5^. This Ibex controller is a small processor with a 2-stage pipeline and uses RV32IMC (Waterman et al., 2014) instruction set (Figure 5).

### 3.2. Neuron processing elements (NPEs)

The SENECA core includes an array of neuron processing elements (NPEs) that act as physical neurons in Figure 1C. Each NPE contains a small register-based memory and executes a category of instructions. An array of NPEs is forming a SIMD (Single Instruction Multiple Data) type architecture (Flynn, 1972). Instructions to be executed in NPEs are coming from the Loop Buffer. NPEs can get their data from Data Memory (through a wide Data Memory port), RISC-V (by directly writing into their register file), and Loop controller (broadcasting).

The register file inside the NPEs allows for reusing data as much as possible before reading/writing it into the Data Memory. Table 2 shows that accessing the data in NPEs' register file is about 20 × more energy efficient than accessing the Data in the Data Memory (SRAM). For example, in an extreme case where the number of neurons is low^6^, keeping the neuron states inside the NPEs and only reading the weights from Data Memory (avoiding the neuron state read/write) reduces the energy consumption of a synaptic operation from 2.8 to 1.8 pJ^7^.

In neuromorphic applications, the optimized resolution of neuron states and synaptic weights depends on several variables (Khoram and Li, 2018). Therefore, to optimize the memory footprint and access energy, it is crucial that our NPEs support various data types and precision. Currently, NPEs are designed to support 4, 8, and 16 bit data precisions, both for linear and logarithmic quantization (floating point). They also support shared scale factors (Köster et al., 2017; Moons et al., 2017; Jacob et al., 2018; Kalamkar et al., 2019; Coelho et al., 2021). This flexibility allows for the memory-efficient deployment of mixed precision neural networks for inference and on-device adaptation. Each NPE consumes 1.3% of the total area of the core.

### 3.3. Loop controller

The loop controller accelerates part of the controller's task in Figure 1 by orchestrating the time-multiplexing of physical neurons and generating a Data Memory address for the Data Memory access. Loop controller has an important role in improving the energy efficiency of SENECA.

As mentioned, NPEs do not implement a specific neuron model. They only execute special operations, which are common among many neuron models. A neuron/synapse/learning model can be built by sequential execution of a few instructions, called microcode. The loop controller sends the microcode to the NPEs in a “for-loop” style to process events. Therefore, the Loop controller is optimized to execute nested loops. Executing loops using the loop controller is 100 × more energy efficient compared to the RISC-V.

Loop buffer in Figure 4 is a small register-based memory to store a few microcodes. Each microcode is called to process a type of event (for example, neuron update or neuron threshold evaluation).Micro-Code 1 shows an example of a micro-code. The instructions are located inside the loop buffer memory. The loop controller dispatches the instructions to NPEs (same instructions for all NPEs) one by one and the corresponding address to the Data Memory. The codes executed in the loop buffer have a special structure in the form of nested loops. This format is optimized for executing neural networks and is flexible enough for executing the core of all neural network algorithms.

Processing of an event requires a set of information that RISC-V provides to the Loop controller in the form of Tasks, queued in the Task FIFO. Since the loop buffer holds several micro-codes, it must be clear which micro-code should be executed. Each task also contains one or more addresses (e.g., weight address in Micro-Code 1). Task FIFO allows RISC-V to push future tasks for processing without waiting for the current task to be completed. The micro-code will execute in parallel in all the NPEs. Every instruction executes in one cycle (pipelined); therefore, the execution of a micro-code can take several hundred cycles.

**Example of a micro-code for a fully connected layer, with 2,048 neurons.**


While(task exists in the Task FIFO) //process
   events 
  //initialized by RISC-V
  State_Addr = 0x100120
   //Copy the weight address from the task FIFO
   Weight_Addr = TASK_FIFO_ADDR
   //update 256x8 neurons (8=number of NPEs)
   for (i=0, i <256, i++)
       for (j=0, j <32, j++)
           R1 = DMEM [State_Addr*i+j] //Load 8
                   neuron states
           R2 = DMEM [ Weight_Addr*i+j] //Load 8
                   weights
           R1 = R1 + R2 //8 Accumulation
           DMEM [State_Addr*i+j] = R1 //Store the 8
                       states

### 3.4. Event generator

As mentioned, due to axon time-multiplexing, every time a neuron fires, we need to convert its output to a packet of data. The event generator performs this task after receiving the corresponding instruction from the Loop controller. This block inspects one of the internal registers of NPEs. Depending on a predefined condition, it generates a packet (event) containing a unique address (source neuron ID) and an optional value (for graded spikes). The generated events will be collected in a FIFO and provided to RISC-V for further post-processing of events (e.g., adding a core address to it, compression, etc.).

### 3.5. Network-on-chip (NoC)

To connect the neuro-synaptic cores and deliver the spike events, SENECA is using an NoC with a minimal footprint. This NoC supports multicasting (source-based addressing) and variable-length packets (needed for compression). Multicasting and event compression features can help to reduce the total communicated bits.

**Figure 6:**
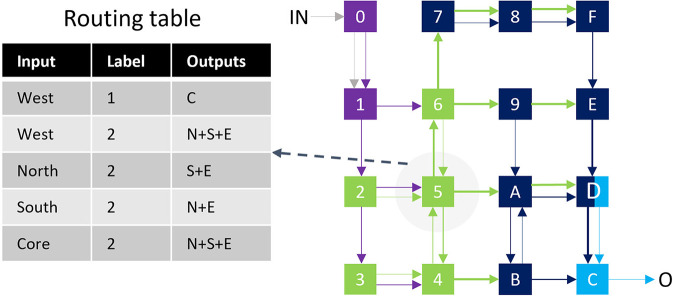
Example of mapping a four-layer neural network (Color coded in cores and links) in a 16-core chip. The routing table for core-5 is shown on the left.

The multicasting feature is implemented using filters stored in a register-based routing table inside each router (a similar approach to Furber et al., 2014). Every filter entry contains three fields: *Input*, *Lable*, and *OutputPorts*, which define the output port for each input event. Figure 6 illustrates a mapped neural network and the corresponding routing table for one of the cores. Even though increasing the number of filters increases the routing flexibility, a small set of filters is sufficient for many neural networks with structured connectivity. For non-structured connectivity types (like millions of randomly connected neurons), a less optimum routing might be used due to limited filtering capacity.

### 3.6. Shared memory pre-fetch unit (SMPU)

A SENECA core may extend its local Data Memory by using denser and larger shared memory blocks, as shown in Figure 4. The primary motivation behind this decision is to use a different memory technology that allows us to improve the area efficiency of a core. Shared memory can be implemented either on-chip using newer and denser memory technologies (e.g., STT-MRAM) or off-chip using, for example, a 3D stacked memory technology (Beyne et al., 2021; Sheikh et al., 2021; Bamberg et al., 2022). Shared memory is optional and will only be used if the local data memories are not enough to store the parameters. Also, non-volatile shared memory allows to power off the volatile memories of a core during low activity times to reduce leakage power. It is important to note that, unlike conventional GPU architectures, SENECA's shared memories are not supposed to be used to communicate between processing cores.

Shared Memory Pre-fetch Unit (SMPU) is an optimized DMA that enables efficient shared memory access through a direct link to the arbiter of the shared memory (Figure 4). Since shared memory is far from the neuro-synaptic cores, each data transfer will cost more energy and latency (Table 2). SMPU can hide the extra latency by pre-fetching the required parameters for events that are waiting in the queue.

## 4. Analysis and results

**Figure 7:**
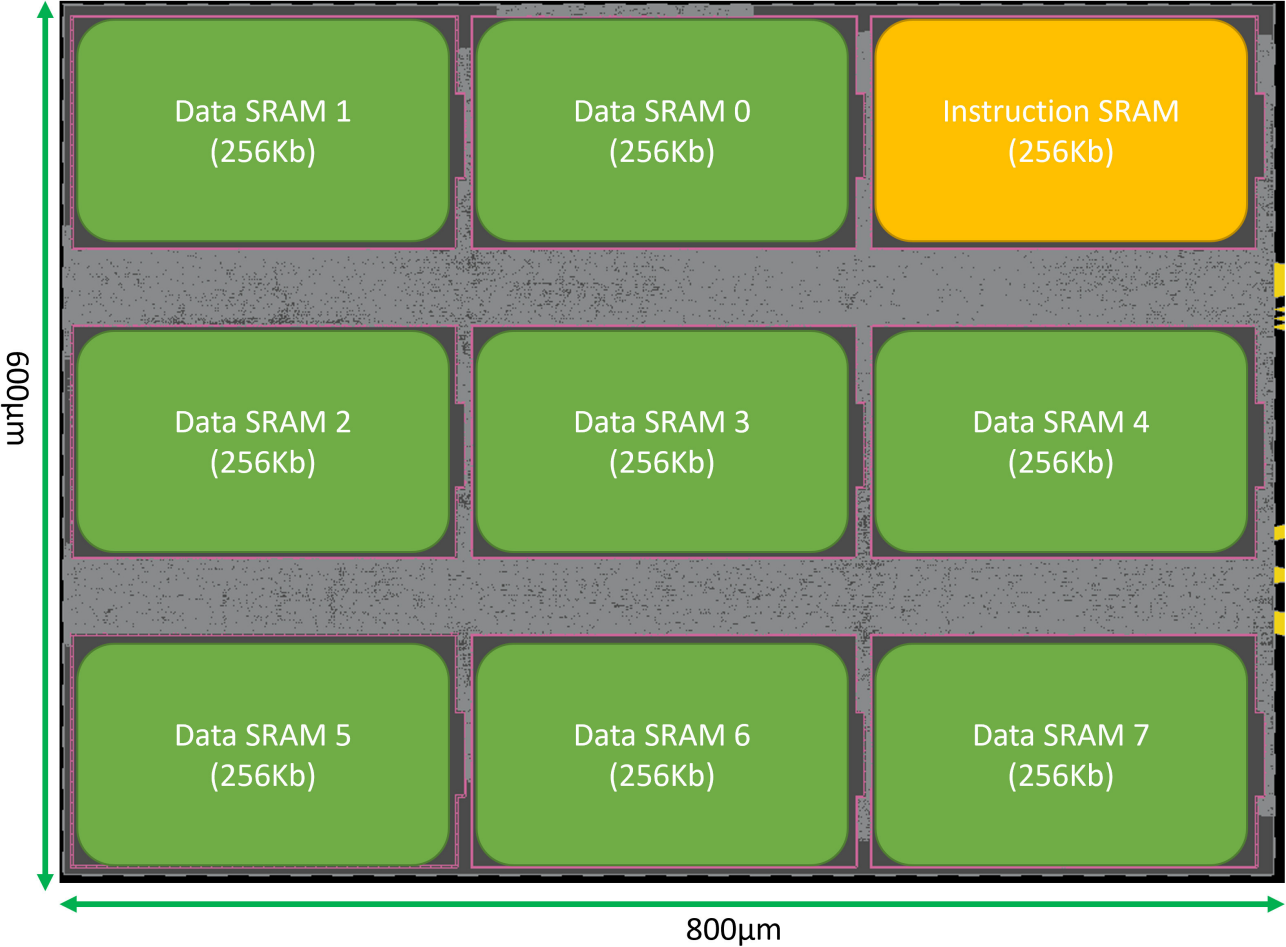
Snapshot of the physical implementation of a SENECA core in GF-FDX22nm technology using the Cadence Innovus implementation system. Green boxes show the banks of Data memory. The orange box is the instruction memory, and the distributed gray cells are the standard logic.

SENECA core can be synthesized with various parameters. Table 6 shows the parameters and their default values used in this paper for synthesis. This section provides the area measurements of a SENECA core. This information can be used to estimate the area for a scaled-up system with an arbitrary number of cores. Since optimizing the leakage power is important for neuromorphic processors, we decided to target FDX-22nm technology from Global Foundries (GF-22 nm) as an ultra-low-leakage technology node. Figure 7 shows the physical implementation of a single SENECA core. Table 7 shows the breakdown of area consumption, and Table 1 compares it with other neuromorphic processors. SENECA has a high area efficiency which comes from the flexibility in mapping, logic time-multiplexing and using hierarchical memory architecture.

**Table 6:** Available synthesis parameters in a SENECA core and their default value, used in this paper.

**Parameter**	**Default value**
Number of NPEs	8
Per NPE register file size	64 × 16 b
Loop buffer register file size	128 × 23 b
Data memory size	2 Mb
Instruction memory size	256 Kb
Event generator FiFo size	128 × 23 b
NoC input FiFo size	128 × 32 b
NoC output FiFo size	32 × 32 b
Loop buffer event address FiFo size	16 × 23 b
Loop buffer event data FiFo size	16 × 16 b

**Table 7:** Area consumption (cell area plus wiring) of one neuro-synaptic core and its components in the GF-22 nm technology node, using Cadence Genus tool.

**Module**	**Cell count (*k*)**	**Area(*kμm*^2^)**	**Area (%)**
RISC-V	11	10.9	2.3
SMPU	1.7	2.1	0.4
NoC	9.8	12.1	2.6
RV peripherals	2.9	2.4	0.5
NPE	5.6	6.3	1.3
Event generator	7.3	9.7	2.1
Loop buffer	8.9	10.5	2.2
Inst Mem	1 × 256 kb	41.2	8.7
Data Mem	8 × 256 kb	330.7	70
Total core	92.6	472.4	100

We configured this neuro-synaptic core to have 8 NPEs, 22 kb of register-based memory, 2 Mb of Data Memory, and 128 kb of instruction memory with 500 MHz clock frequency.

### 4.1. Instruction level benchmarking

As mentioned, NPEs can execute various instructions. Each instruction execution requires the engagement of Loop Buffer, NPEs and possibly Event Generator and Data Memory. We have performed a detailed instruction-level energy measurement of a SENECA core and report the average energy consumption of some of the NPE instructions in Table 8^8^. The pre-silicon energy breakdown includes the power consumption of NPE plus all the modules needed to execute the instruction in NPEs. However, since those blocks are shared between 8 NPEs, their contribution in total energy per instruction is divided proportionally. The results are measured by running each instruction 8, 000 times with random data using the Cadence JOULES (time-based mode), an RTL level power measurement tool (within 15% of signoff power; Cadence, 2021), and with the GF-22 nm FDX technology node in the typical corner (0.8*v* and 25*C*, no back-biasing). Reported energy consumption includes the total (both dynamic and static) power consumption of one SENECA core while executing the instruction. The leakage power for the complete SENECA core is around 30μ*W* (0.06 pJ in a 2 ns clock cycle).

**Table 8:** Energy consumption breakdown for various instructions executes in NPEs.

**Instruction**	**Description**	**Energy of**	**Energy of**	**Energy of**	**Total Energy**
		**computation**	**each NPE**	**loop buffer**	**per instruction**
ADD/SUB/MUL	FP16 Arithmetic ops.	0.5	1.3	0.9	1.4
	2xINT8b Arithmetic ops.	0.3	1.1	0.9	1.2
GTH/MAX/MIN	FP16 Compare ops.	0.3	1.1	0.9	1.2
EQL/ABS		0.2	1.0	0.9	1.1
AND/ORR	16b Bit-wise ops.	0.2	1.0	0.9	1.1
SHL/SHR		0.3	1.1	0.9	1.2
I2F	Data type cnv.	0.3	1.0	0.9	1.1
RND		0.6	1.3	0.9	1.4
**Instruction**	**Description**	**Energy of**	**Energy of**	**Energy of**	**Total energy**
		**Data-Mem-16b**	**each NPE**	**loop buffer**	**per instruction**
MLD	16b Data Mem load	2.9	0.6	1.6	3.7
MST	16b Data Mem store	3.5	0.2	1.6	3.9
**Instruction**	**Description**	**Energy of**	**Energy of**	**Energy of**	**Total energy**
		**event generator**	**each NPE**	**loop buffer**	**per instruction**
EVC	Event capture	0.6	0.4	0.5	0.5
	+ per generated event	+1.1	+0	+0	+1.1
**Instruction**	**Description**	**Energy of**	**Energy of**	**Energy of**	**Total Energy**
		**RV+Peripheries**	**Inst-Mem-32b**	**Data-Mem-32b**	**per instruction**
RISC-V Ops	Averaged per instruction	5.9	5.7	0	11.6
	+ Data Mem access			+10	+10
NOC	Per 32b event transmission	–	–	–	2

The energy of the computation is part of the NPE's energy, consumed by the involved compute logic inside the NPE. All the numbers are in *pJ*.

Reported energy numbers in Table 8 are measured considering the pessimistic scenario of switching and randomness. In practical scenarios (also shown in the next sections), the instruction power consumption is less than the reported numbers.

As seen in Table 8, the energy consumption of the computing unit (the involved part of the ALU inside the NPE which executes the computation) is a small part of the total energy consumption. To execute an instruction like ADD, it is required to access three registers, which is as power expensive as the instruction itself. By looking into the energy consumption of Data Memory access, it can be seen that the location and resolution of data can significantly change the overall power consumption of an algorithm. Using Table 8, it is possible to estimate the energy consumption of a synaptic operation for various neuron models, parameter resolutions and memory mapping.

To update an Integrate-And-Fire neuron (Abrahamsen et al., 2004) and perform one synaptic operation in its simplest form, it is required to load the neuron state and synaptic weight from memory, add the synaptic weight to the neuron state and store the updated neuron state back. This synaptic operation can be done with the first implementation of Micro-Code 2 and consumes 12.7 pJ. If we use low precision parameters (4b weight and 8b state) and then perform integer operation, as shown in the second implementation of Micro-Code 2, the cost of synaptic operation will drop to 5.6 pJ. The cost of synaptic operation can drop even further with “spike-grouping,” where we reuse the loaded neuron state by processing a group of spikes together. For example, in the third implementation of Micro-Code 2, we load each neuron state once and update it with a group of four spikes before storing it back in the memory, resulting in 2.8 pJ per synaptic operation. Spike-grouping implementation assumes that several neurons in the previous layer fire simultaneously, which is common.

**Integrate-and-fire neuron, instruction level benchmarking.**


  First implementation (1 SOP) 
R1 = DMEM[State_Addr*i+j]     //3.7pJ 
R2 = DMEM[Weight_Addr*i+j]     //3.7pJ 
R1 = R1 + R2   //1.4pJ 
DMEM[State_Addr+i] = R1     //3.9pJ 
    //Total = 12.7pJ 
  Second implementation, Low Precision (4 SOPs) 
R1 = DMEM[State_Addr]     //2*states 3.7pJ 
R2 = DMEM[State_Addr+1]     //2*states 3.7pJ 
R3 = DMEM[Weight_Addr]     //4*weights 3.7pJ 
R1 = R1 + R3     //2*Int_ADD 1.2pJ 
R3 = R3>>8     //Shift 1.2pJ 
R2 = R2 + R3     //2*Int_ADD 1.2pJ 
DMEM[State_Addr] = R1     //2*states 3.9pJ 
DMEM[State_Addr+1] = R2     //2*states 3.9pJ 
    //Total = 22.5pJ (5.6pJ per SOP) 
  Third implementation, Low Precision + spike-grouping (16 SOPs) 
R1 = DMEM[State_Addr]     //2*states 3.7pJ 
R2 = DMEM[State_Addr+1]     //2*states 3.7pJ for(i=0; i <4, i++) 
    R3 = DMEM[Weight_Addr(i)]     //4*weights 3.7pJ 
    R1 = R1 + R3     //2*Int_ADD 1.2pJ 
    R3 = R3>>8     //Shift 1.2pJ 
    R2 = R2 + R3     //2*Int_ADD 1.2pJ 
DMEM[State_Addr] = R1     //2*states 3.9pJ 
DMEM[State_Addr+1] = R2     //2*states 3.9pJ 
    //Total = 15.2pJ+4*7.3 (2.8pJ per SOP)

### 4.2. Algorithms level benchmarking

Instruction level benchmarking can provide a fast estimation of the energy cost of an application composed of many instructions. However, it cannot accurately predict the overhead costs and the timings in more complicated scenarios. To perform a more accurate benchmarking, we implemented a few examples of the most common neural network layers and learning algorithms to measure their energy and execution times.

#### 4.2.1. Event-driven fully-connected processing

Fully-connected computations on all-to-all connections between input neurons and output neurons form the basis of many neural network architectures, including multilayer perceptron (MLP), recurrent neural networks (RNN), convolutional neural networks (CNN), and more recently, transformers, and MLP-Mixers (LeCun et al., 2015; Vaswani et al., 2017; Tolstikhin et al., 2021). To reduce the computational cost, existing algorithms utilize input sparsity with binary spikes from SNNs (Zambrano et al., 2019) and graded spikes from DNNs (Yousefzadeh et al., 2019; Kurtz et al., 2020). In this section, we implement the event-driven processing of a fully-connected layer in SENECA and benchmark its performance with binary and graded spikes, low-precision parameters, and spike-grouping.

**Figure 8:**
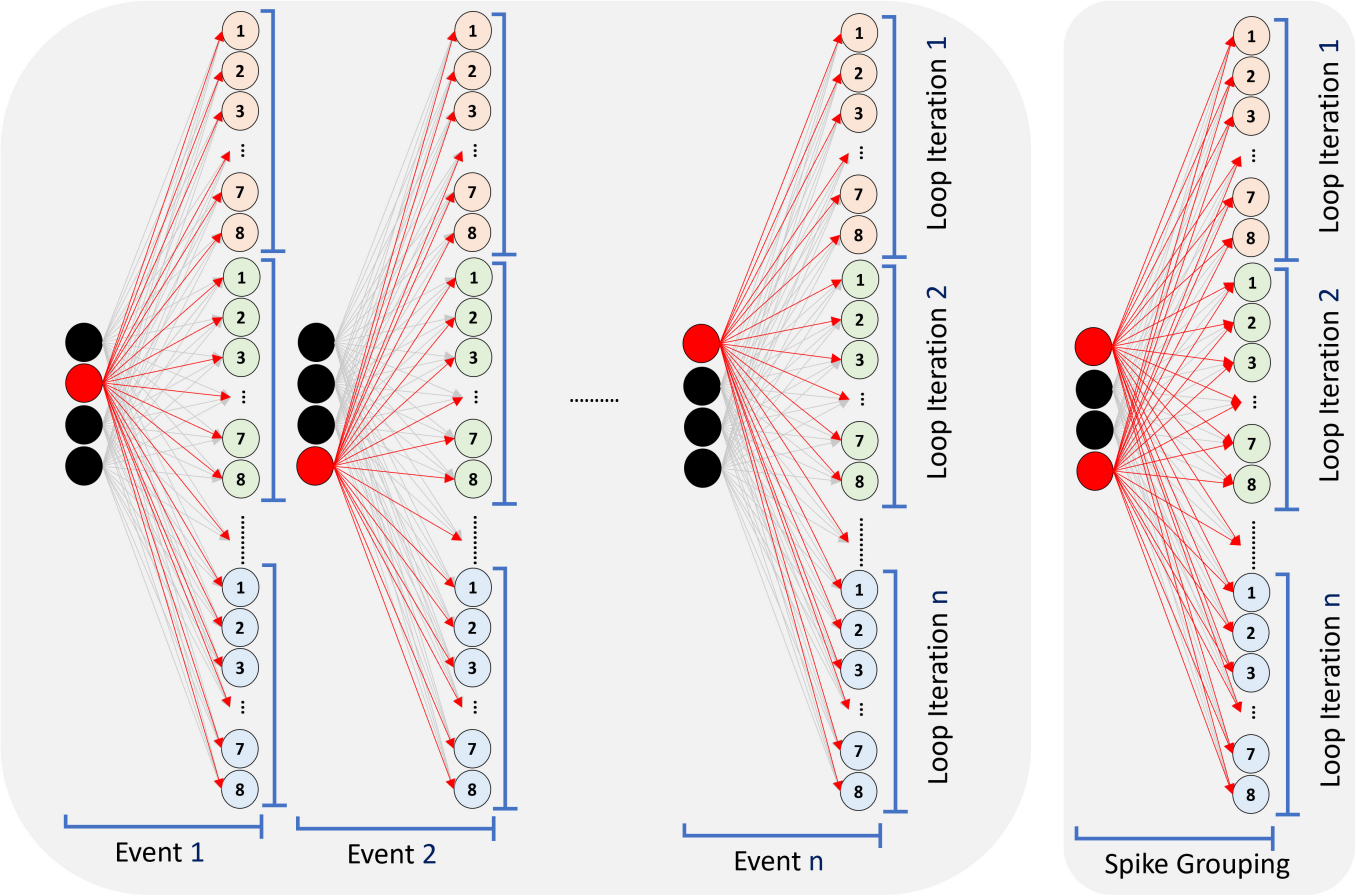
Processing a fully connected layer in an event-driven model. Processing each event requires reading all the synaptic weights from Data Memory, Reading neurons' state (membrane), updating them and writing them back. Since the core has eight NPEs, only eight neurons will be updated in each internal loop iteration. Spike-grouping (right) reduces the memory access (read/write) for the neuron states by processing several spikes simultaneously.

Figure 8 illustrates the event-driven processing in SENECA that can exploit the sparsity in the inputs for fully-connected computation. Each incoming input spike is processed in order by adding the corresponding synaptic weight to all post-synaptic neurons. For graded spikes, the graded value is multiplied by the synaptic weight before adding to the neuron state. For spike-grouping, multiple input spikes are integrated into the neuron state in the same iteration, reducing the neuron state memory access.

**Figure 9:**
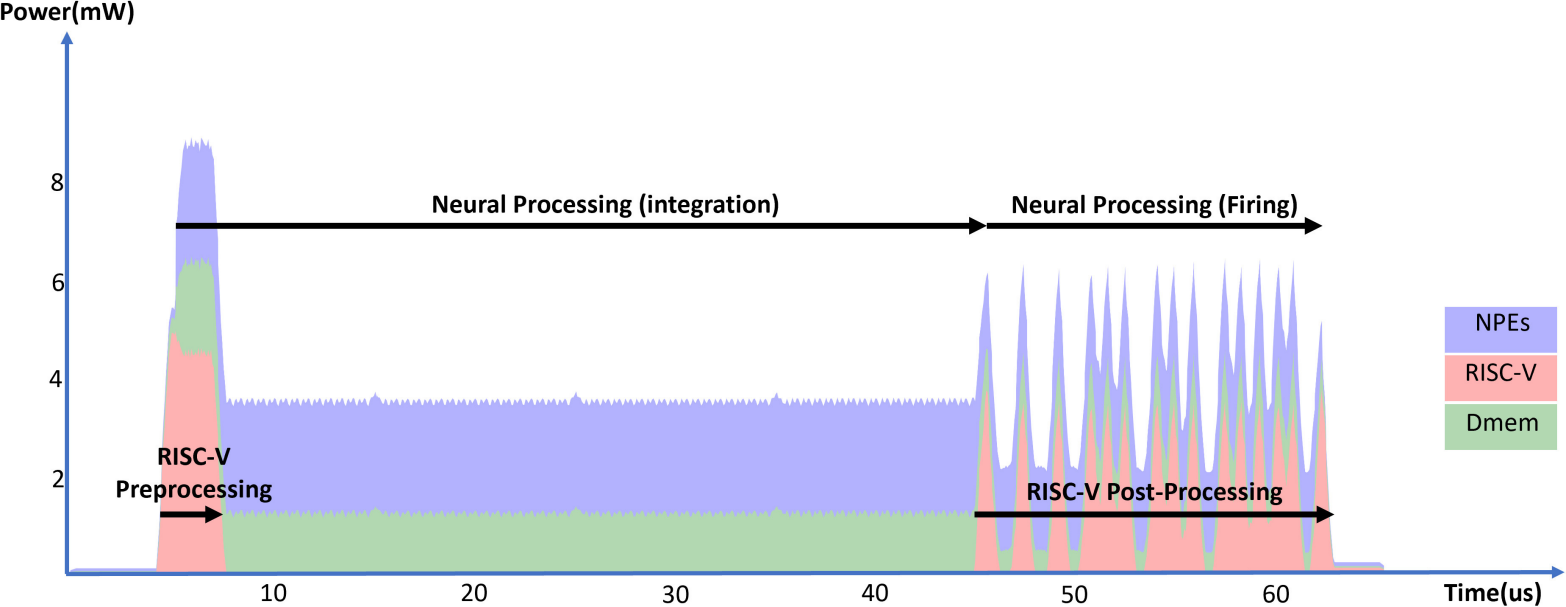
Total power consumption of a SENECA core in time when processing 16 incoming spikes in a fully connected layer and generating (fire) 16 output spikes.

Generally, inference of a neural network layer in SENECA consists of three phases: preprocessing, integration, and firing (see Figure 9). In the preprocessing phase, RISC-V preprocesses the input spikes by finding the local memory addresses of the weights and the output neuron states based on the input spikes' source address. The loop buffer starts executing the neural integration phase as soon as RISC-V finishes pre-processing the first spike. After processing all spikes and at the end of the time-step, the firing phase will be first executed inside the NPEs, which results in generated events inside the event generator. RISC-V then reads the generated event, computes, and attaches the extra information through post-processing before sending out a compressed spike packet. The RISC-V preprocessing time depends on the number of incoming spikes, while the RISC-V post-processing time depends on the number of generated events. The operation time of NPEs depends on the number of spikes and the number of neurons in the layer.

Table 9 shows the time/energy measurements of the several implementations/mappings of the fully connected layer. In all the experiments, 16 input spikes are processed, and 16 output spikes are generated. The fully connected layer contains 4, 000 neurons. In the “Graded Spike” experiment [Baseline], the spike value, weights and neuron states are 16b. The second experiment shows 6.1% energy reduction when using binary spikes instead of graded (floating point) spikes. In the “spike-grouping” experiment, we process four graded spikes together, as explained in Micro-Code 2, which results in 47.0% energy reduction over the baseline. The fourth experiment combines binary spikes and weight quantization. In this experiment, we use binary spike, 4b weights and 8b neuron states, allowing us to use the integer ADD operations. Using quantization and binary spikes results in a 52.7% energy reduction over the baseline. By mixing binary spike, quantization, and spike-grouping, we reduce the energy consumption of baseline implementations by 80.7%.

**Table 9:** Experimental results for fully connected layer.

	**Time**	**RISC-V**	**NPEs**	**Dmem**	**Total core**	**Energy per**
**Experiment**	**(*μS*)**	**energy (*nJ*)**	**energy (*nJ*)**	**energy (*nJ*)**	**energy (*nJ*)**	**SOp (*pJ*)**
[Baseline]						
Graded spikes	228	11.7	423.3	434.0	908.8	14.2
Binary spikes	179.9	8.4	288.7	525.8	853.1	13.3
Spike-grouping	121.8	13.4	291.8	155.0	481.9	7.5
Binary Spike						
+Quantization	109.2	10.7	143.5	254.8	429.5	6.7
Binary Spike						
+Quantization						
+Spike-grouping	57.7	10.5	100.4	52.9	175.5	2.7

RISC-V energy includes RISC-V and its instruction memory. NPEs energy includes all NPEs, Loop buffer, and Event-generator blocks. Synaptic operation (SOp) energy is calculated by dividing the total core energy by 64 k (4,000 neurons, each processing 16 spikes).

Using binary spikes reduces the number of computations (skipping the spike-weight multiplication). On the other hand, spike grouping reduces the amount of memory access by reusing the neuron states in the NPEs' register file. As seen in Table 9, memory access optimization has a more significant effect on energy and processing time. Weight quantization reduces both computational cost and memory access. However, neural networks lose accuracy when quantized. Since it is possible to trade off the number of parameters, sparsity, and accuracy of a neural network, it is not known in priory if a quantized network is the most hardware efficient one (Kim et al., 2022). SENECA architecture provides enough mapping flexibility for neural architecture search (NAS) approaches to co-optimize algorithm accuracy and hardware performances (Benmeziane et al., 2021; Chitty-Venkata and Somani, 2022).

#### 4.2.2. Event-driven convolutional neural layer processing

Spiking convolutional neural networks have been widely used in neuromorphic computing for event-based processing (Yousefzadeh et al., 2017b; Kheradpisheh et al., 2018; Negri et al., 2018; Patino-Saucedo et al., 2020; Lv et al., 2023). The convolutional neural layer consists of a sequence of fully-connected operations on overlapping local regions of the input space using shared weights. Efficient event-driven convolutional processing requires weight reuse for memory efficiency and sparse input spikes for computational efficiency (Yousefzadeh et al., 2015). Compared with the fully-connected processing presented in Section 4.2.1, the event-driven convolutional operation requires a more complex pre-processing and post-processing. In this section, we implement the event-driven processing of a convolutional neural layer in SENECA and benchmark the hardware performance of the processing.

**Figure 10:**
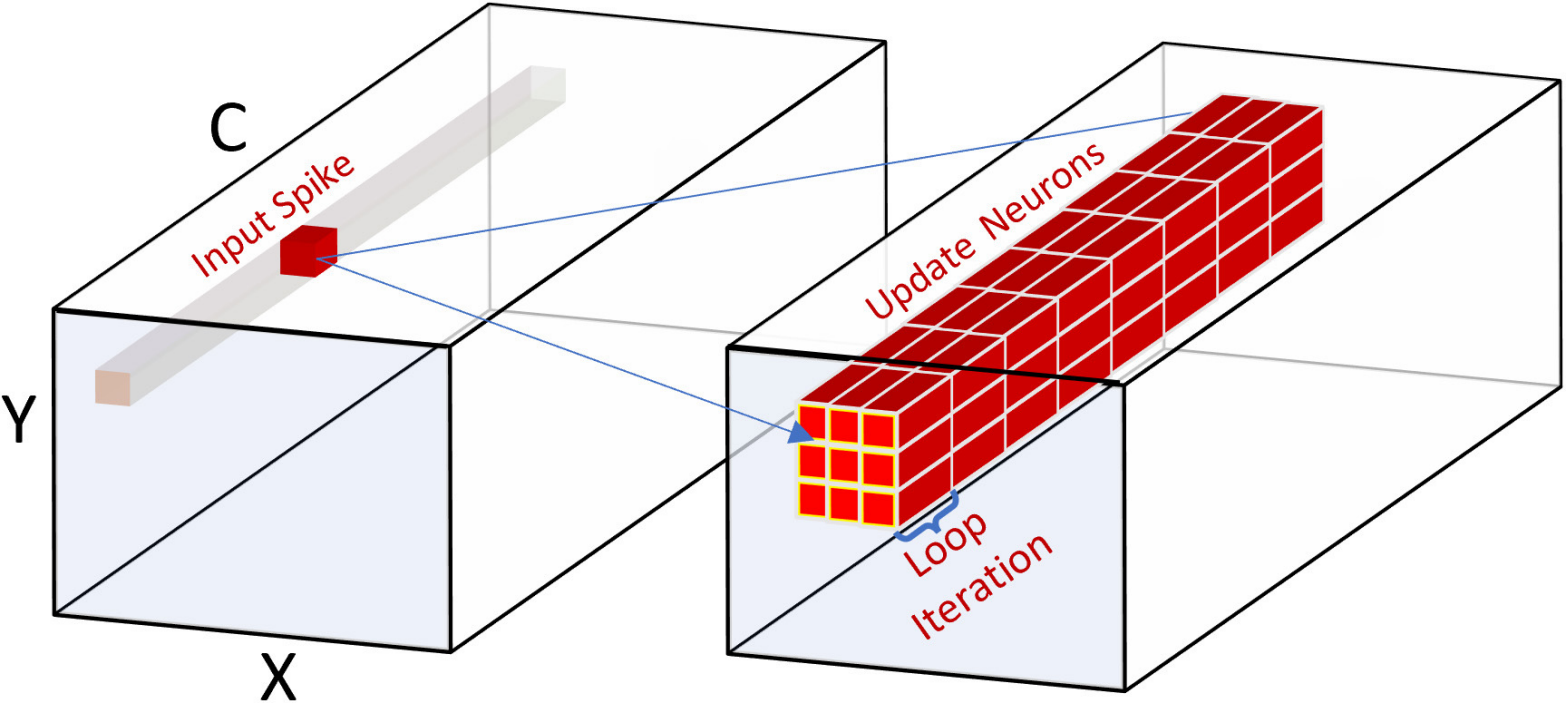
Processing a spike in a convolutional layer. The neurons in the projection field will be updated after receiving the input spike shown in this figure.

Event-driven convolutional processing directly integrates the input spike to post-synaptic neurons in the spike's projection field without waiting for all spikes to arrive. Figure 10 illustrates the event-driven convolutional neural layer processing in SENECA, in which a single incoming spike is integrated into the post-synaptic layer with 2D convolutional connectivity. In this case, each spike carries information about the coordination of the source neuron and its channel number from the previous layer. Based on these coordinates, RISC-V calculates the projection field's start address and the corresponding shared weights' address to support NPE processing. As a result, the RISC-V operations in the convolutional layers are slightly more complex than the fully connected layers.

**Figure 11:**
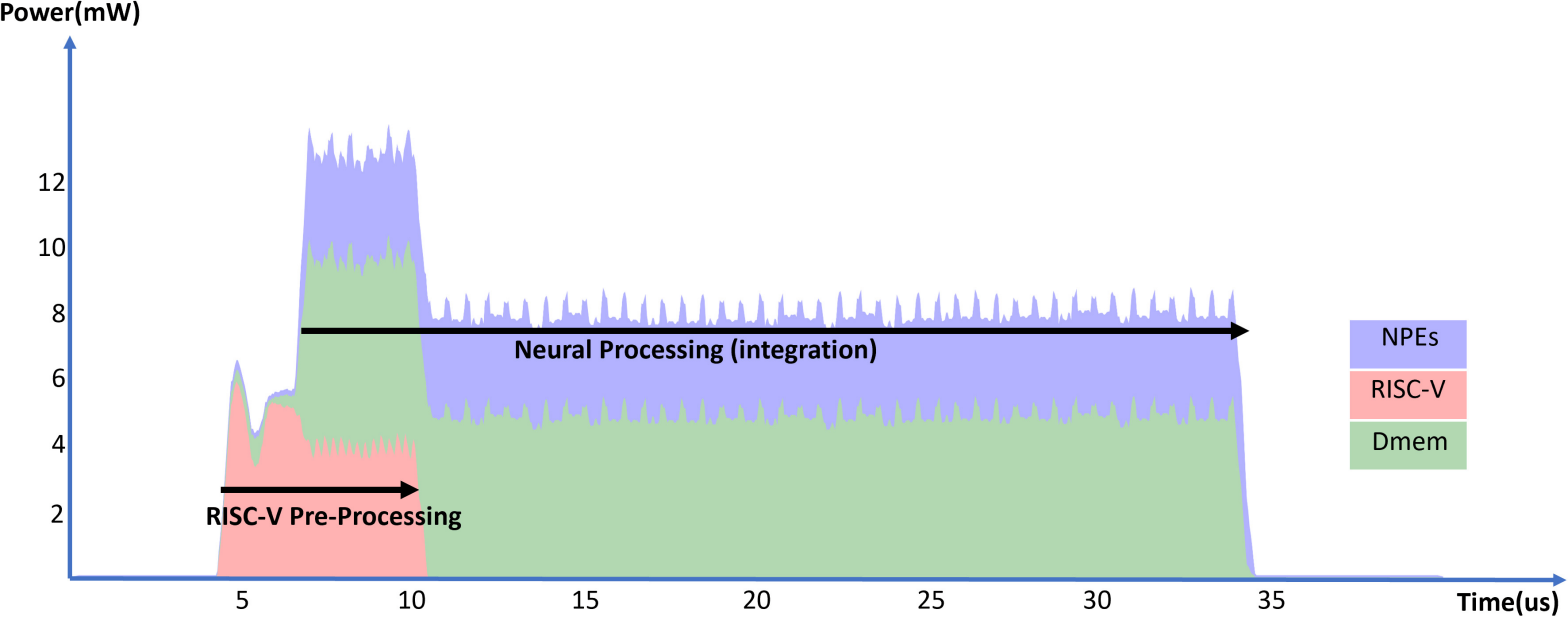
Total power consumption of a SENECA core in time when processing 16 spikes in a convolutional layer.

Figure 11 and Table 10 show the energy measurements of the convolutional layer implementation. In the experiment, we measured a convolutional layer with 128 channels processing 16 input spikes from the previous layer. This experiment uses BF16 values for input spikes, weights, and neuron states. By using the 3 × 3 kernel sizes, each input spike updates a projection field of 3 × 3 × 128 neurons.

**Table 10:** Experimental results for convolutional layer.

**Time**	**RISC-V**	**NPEs**	**Dmem**	**Total core**	**Energy per**
**(*μS*)**	**energy (*nJ*)**	**energy (*nJ*)**	**energy (*nJ*)**	**energy (*nJ*)**	**SOp (*pJ*)**
29.6	12.1	75.4	126.6	221.9	12.0

RISC-V energy includes RISC-V and its instruction memory. NPEs energy includes all NPEs, Loop buffer, and Event-generator blocks. Synaptic operation (SOp) energy is calculated by dividing the total core energy by 18.4 k (128 × 3 × 3 neurons, integrating 16 spikes). In this experiment, the firing of neurons is not included.

The incoming 16 spikes are from the same (X, Y) location but various channels. This is very common in event-driven convolutional processing since all the neurons in different channels in an (X, Y) location update and fire simultaneously. We exploit this feature with the following techniques to further reduce the cost of communication and pre-processing:

- Creating a compressed packet of spikes by sending the source (X, Y) address of all the spikes only once in the header, followed by the (Channel, Value) of each spike.
- Processing the (X, Y) location in the RISC-V only once to find the neuron states in the projection field.

The energy measurements did not include the firing phase of the neurons. The event-driven convolutional processing in SENECA can support depth-first CNN, which spontaneously fires neurons that receive all inputs in its receptive field (Goetschalckx et al., 2022; Lv and Xu, 2022; Symons et al., 2022). This can avoid keeping the state of all neurons in the memory and results in lower latency for CNN processing compared to the layer-wise synchronized firing in existing neuromorphic hardware (Hwu et al., 2017; Massa et al., 2020). Although this is out of the scope of the paper, the flexibility of the RISC-V controller makes it possible to have efficient depth-first spike generation in the future.

#### 4.2.3. Recurrent on-device learning with e-prop

Recurrent spiking neural networks (RSNN) consist of recurrent connections and spiking neurons. With sparse recurrent spikes on top of stateful neurons, RSNN learns temporal information from sequence data better than vanilla SNN (Yin et al., 2021; Kumar et al., 2022). Training of RSNN using backpropagation-through-time (BPTT) requires unrolling the network on the time dimension and performing temporal backpropagation (Bellec et al., 2018; Wu et al., 2018), which is memory and computation intensive. To make RSNN learning suitable for edge applications, alternative online-learning algorithms have been proposed to compute gradients without temporal unrolling and backpropagation (Bellec et al., 2020; Tang et al., 2021; Bohnstingl et al., 2022). The e-prop algorithm has demonstrated state-of-the-art online recurrent learning performance (Bellec et al., 2020; Traub et al., 2022). As the core component of e-prop, the eligibility trace computes the local gradients of synaptic weights in real-time during forward propagation. In this section, we implement the eligibility trace computation of e-prop in SENECA and benchmark the algorithm's performance for RSNN learning.

The e-prop eligibility trace *e*_*ij*_ computes the local gradient $\frac{dz_{j}}{dW_{ij}}$ of the synaptic weight *W*_*ij*_ with respect to the spike output *z*_*j*_ of the post-synaptic layer. By employing the past-facing perspective of recurrent learning, e-prop approximates the local gradient using a Hebbian-like learning rule combining pre and post-synaptic information. When using RSNN with leaky-integrate-and-fire (LIF) neurons, the eligibility trace is computed as follows,


(1) $$\begin{array}{l} trace\left\{z_{in,i}\right\}\left[k\right]=trace\left\{z_{in,i}\right\}\left[k-1\right]+\beta \cdot z_{in,i}\left[k\right] \end{array}$$

(2) $$\begin{array}{l} e_{ij}\left[k\right]=trace\left\{z_{in,i}\right\}\left[k\right]\cdot h\left(v_{j}\left[k\right]\right) \end{array}$$

where *trace* is the input trace of pre-synaptic spikes *z*_*in, i*_, β is the leak of the LIF neuron model, *v*_*j*_ is the neural state, *h* is the surrogate gradient function that estimates the non-differentiable spiking function, and *k* is the timestep.

We implemented the e-prop eligibility trace computation with an RSNN layer in SENECA. The RSNN layer implementation uses the same synaptic integration phase as the fully-connected layer presented in Section 4.2.1 using graded spikes. Recurrent spikes from the previous timestep are buffered and then processed in the same way as the input spikes. Additional pre and post-synaptic information needs to be prepared to compute the eligibility trace, including the input trace and the output surrogate gradient. For memory efficiency, we compute the input trace separately for each input dimension instead of repeating the computation for each synaptic weight. The surrogate gradient computation is fused into the firing phase to avoid additional memory access. Here, we used a rectangular function introduced in Wu et al. (2018) as the surrogate gradient function. The eligibility trace matrix is the outer product of the input trace vector and the surrogate gradient vector. To compute this outer product, we feed the input trace as events to the NPEs and parallelize the computation on the output dimension.

**Figure 12:**
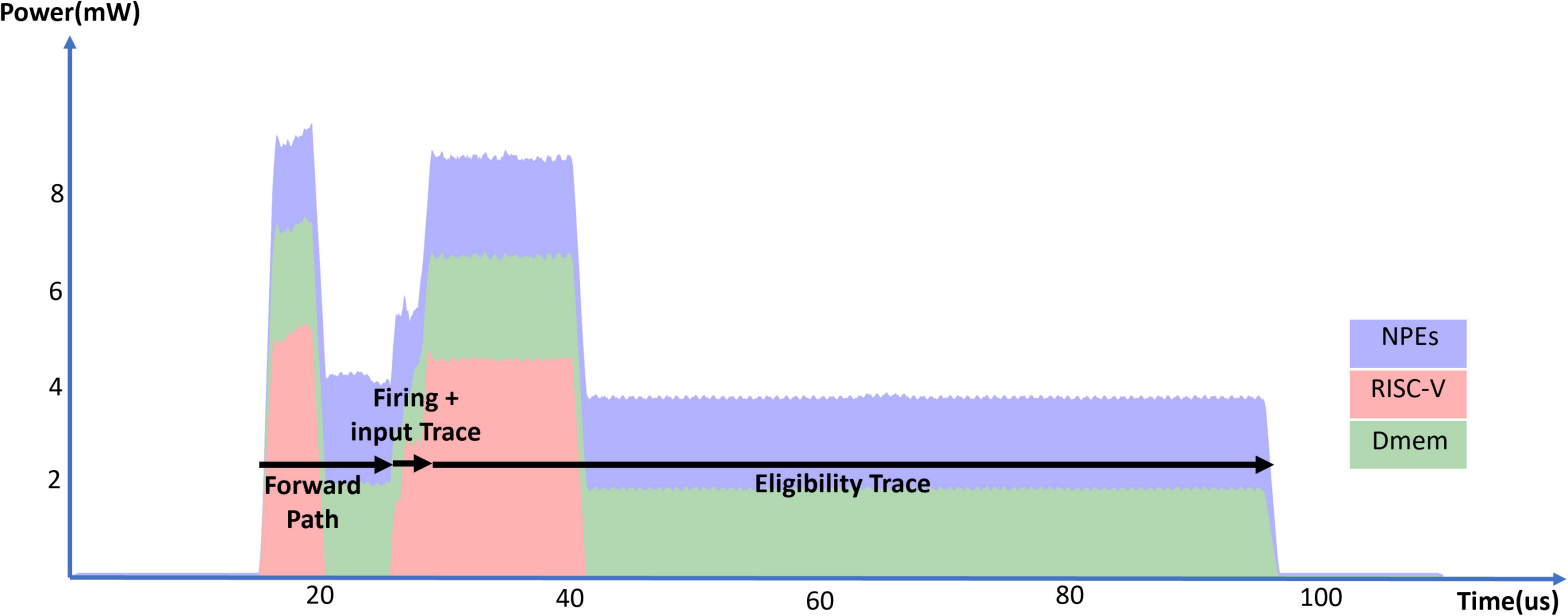
Total power consumption of a SENECA core in time when executing the RSNN layer equipped with the e-prop online learning.

Figure 12 and Table 11 show the energy measurements of the eligibility trace computation with an RSNN layer in SENECA. We constructed an RSNN layer with 32 input neurons and 128 output neurons. Since the RSNN has fully connected recurrent connections, the input dimension to the output neuron is 160. The memory overhead of e-prop consists of the input traces (160 × 16*b*), post-synaptic surrogate gradients (128 × 16*b*), and the eligibility traces (160 × 128 × 16*b*), which roughly doubles the memory requirement of the inference-only RSNN layer. The RSNN layer processes 16 input events and eight recurrent events, and generates 16 output events. As shown in Figure 12, the computation has four phases: RSNN forward path, RSNN firing, input trace update, and eligibility trace update. Table 10 shows the detailed times and energy consumption of each phase. Compared to the fully-connected baseline in Table 8, the e-prop algorithm introduces around 30% overhead on each synaptic operation in the RSNN forward computation (14.1 vs. 18.3 pJ per SOp). This overhead mainly comes from the input spike buffering on RISC-V required for the input trace computation. Due to the dense vector outer product iterating every synaptic weight, the eligibility trace matrix update is the most time and energy-costly phase in our implementation. The cost of this phase can be reduced by exploiting the sparsity in the vectors using the event-driven processing of SENECA.

**Table 11:** Experimental results for e-prop with RSNN.

	**Time**	**RISC-V**	**NPEs**	**Dmem**	**Total core**	**Normalized**
**Algorithm phase**	**(*μS*)**	**energy (*nJ*)**	**energy (*nJ*)**	**energy (*nJ*)**	**energy (*nJ*)**	**energy (*pJ*)**
Forward path	10.5	13.4	18.8	20.7	56.2	18.3/SOp
Firing	0.9	1.3	2.0	0.8	4.5	35.0/Output
Input trace	1.1	2.3	0.9	1.1	4.7	29.3/Input
Eligibility trace	68.2	21.8	117.7	127.8	289.7	14.1/Weight

RISC-V energy includes RISC-V and its instruction memory. NPEs energy includes all NPEs, Loop buffer, and Event-generator blocks.

Even though the deployed algorithm can be further optimized for SENECA (for example, by quantization, sparsification, and spike grouping), it demonstrates the capability of SENECA to execute such a complex pipeline efficiently. Due to the algorithm's popularity, e-prop and its close variants have been benchmarked on several other neuromorphic processors (Tang et al., 2021; Frenkel and Indiveri, 2022; Perrett et al., 2022; Rostami et al., 2022). Those implementations are either forced to be (1) less efficient due to hardware-algorithm mismatch (Tang et al., 2021; Perrett et al., 2022; Rostami et al., 2022) or (2) hard-wired only to execute a limited version of this algorithm (Frenkel and Indiveri, 2022) which cannot adapt to deploy the new and more efficient online learning algorithms (Yin et al., 2021; Bohnstingl et al., 2022).

## 5. Conclusion

In this paper, we introduced the SENECA neuromorphic architecture, a flexible and scalable design that tackles the challenges in neuromorphic engineering. We justified SENECA's design choices by discussing the main trace-offs in the neuromorphic processor design and compared the proposed architecture with existing designs from these perspectives. To demonstrate the efficiency of SENECA, we provided detailed instruction level measurements and algorithm level benchmarking for the few most common algorithms.

The algorithm-level benchmarking shows that the flexibility of SENECA allows us to efficiently map various algorithms without sacrificing energy efficiency. Furthermore, our results show that flexibility increases optimization space and results in more optimized algorithm implementation (e.g., optimized fully-connected processing). The flexibility gives SENECA the potential to outperform a large group of neuromorphic processors when a hybrid of neural network algorithms with on-device learning is required to perform the task (e.g., sensory fusion in automotive applications). This aligns with the trend in the new generation of more flexible neuromorphic architecture compared to the first generations of the same processors to increase the competitiveness of the design in EdgeAI (Mayr et al., 2019; Davis, 2021).

SENECA, like any other neuromorphic chip, is a memory-dominant processor. Memory consumes most of the area and power consumption of the processor. In Table 9, we have shown the performance improvement when saving on the memory access is more significant than saving on the computation. SENECA allows using flexible mapping of neural networks, resulting in high memory efficiency. It also supports a more advanced memory hierarchy, allowing for better scalability and data reuse (For example, spike-grouping in Table 9). For future work, we will look into optimizing memory area and power consumption using new memory technologies and 3D integration. We are looking into competitive Non-Volatile Memories (NVM) with high density (e.g., STT-MRAM) to be used as the on-chip shared memory. NVMs can be several times denser than SRAM when deployed in larger blocks. Having a large shared memory allows us to store multiple specialized neural network models and switch between them in different scenarios. Integrating shared memory with advanced 3D technology allows for reducing the distance between the shared memory and the cores, which reduces power consumption and latency.

Our benchmarking results show that computation in RISC-V is significantly more expensive than in the accelerators (like loop buffer and NPEs). Therefore, we accelerate the most common operations shared by many applications. SENECA provides a test bed to measure various accelerators' performance improvement and area overhead. This gives us the opportunity to constantly evaluate SENECA's performance for new neural network algorithms and look for opportunities to add more accelerated operations to the architecture in the future. In conclusion, the SENECA architecture paves the way for future efficient neuromorphic designs in balancing different trade-offs in neuromorphic engineering to achieve high performance and versatility in neural network applications. The SENECA platform and the tools used in this project are available for academic research upon request.

## Data availability statement

The original contributions presented in the study are included in the article/supplementary material, further inquiries can be directed to the corresponding author.

## Author contributions

Hardware design: G-JS, AY, PD, and ST. Algorithm and software design: MS, GT, KV, YX, and KS. Writing the manuscript: MK, MS, GT, YX, AY, G-JS, ST, KV, and PD. PnR area result of the SENECA core: RB. All authors contributed to the article and approved the submitted version.

## Footnotes

^1^Biological action-potential velocity is less than 120 m/s while the speed of a pulse traveling in a wire is around 2 × 10^8^ m/s.

^2^SRAM is inherently analog, but it is used as an IP with digital IOs.

^3^In the neuromorphic processors, the words “events” and “spikes,” and “neuron activation” are used interchangeably. In this context, Event-driven processing means the processing pipeline is triggered by incoming spikes or non-zero neuron activations.

^4^SENECA stands for “Scalable Energy efficient Neuromorphic Computer Architecture.”

^5^ https://lowrisc.org/

^6^Less than 256 neurons in the current setup.

^7^NPE registers are used to keep neuron states, weights, and event values (if used). In addition, some registers are used to store intermediate values in the micro-code. Therefore, the maximum number of neuron states which can be kept locally depends on the micro-code. If we use half of the NPE registers for neuron states, it is possible to keep 256 neurons in the NPEs of a SENECA core (based on Table 6, 8-NPEs, each with 64 registers).

^8^Extra information for Table 8: **Energy of Computation:** Energy consumption of the involved unit of ALU (inside the NPE), which performs the specific operation. **Energy of each NPE:** This energy number includes the total NPE power consumption, including the Energy of Computation, and energy consumption of the access to the register file in the NPE. **Energy of the Loop buffer:** This column reports the total energy of the loop buffer. Loop buffer is shared between 8-NPEs and enables execution of “eight” instructions. **Energy of the Event Generator:** This column reports the total energy of the event generator. The event generator is shared between 8-NPEs and performs eight register inspections. If a register inspection results in a firing, the event generator consumes extra power per firing. **Energy of Data-Mem-16b/Data-Mem-32b:** This column reports the energy consumption of data memory to access 16b/32b of data. **Energy of RV + Peripherals:** Includes energy consumption of the RISC-V and its peripherals (like the main communication bus). **Energy of Inst-Mem-32b:** This column reports the energy consumption of instruction memory to read a 32b instruction. **Total energy per instruction:** This column includes the energy consumption to execute a single instruction. For neural operations in NPE, it includes the energy of one NPE, plus all the overheads (energy of Data-Mem access and one-eighth of loop buffer/event-generator energy, if involved). For RISC-V energy and NoC, it is simply the summation of all the other columns.

## References

[B1] Abrahamsen J. P. Hafliger P. Lande T. S. (2004). “A time domain winner-take-all network of integrate-and-fire neurons,” in Proceedings of 2004 IEEE International Symposium on Circuits and Systems, Vol. 5 (Vancouver, BC). 10.1109/ISCAS.2004.1329537

[B2] Ahmadi-Farsani J. Ricci S. Hashemkhani S. Ielmini D. Linares-Barranco B. Serrano-Gotarredona T. (2022). A cmos-memristor hybrid system for implementing stochastic binary spike timing-dependent plasticity. Philos. Trans. R. Soc. A 380:20210018. 10.1098/rsta.2021.0018 35658675 PMC9168445

[B3] Akopyan F. Sawada J. Cassidy A. Alvarez-Icaza R. Arthur J. Merolla P.. (2015). Truenorth: Design and tool flow of a 65 mw 1 million neuron programmable neurosynaptic chip. IEEE Trans. Comput. Aided Des. Integr. Circuits Syst. 34, 1537–1557. 10.1109/TCAD.2015.2474396

[B4] Altan A. Aslan Ö. Hacıoğlu R. (2018). “Real-time control based on NARX neural network of hexarotor UAV with load transporting system for path tracking,” in 2018 6th International Conference on Control Engineering & *Information Technology (CEIT)* (Istanbul), 1–6. 10.1109/CEIT.2018.8751829

[B5] Amir A. Taba B. Berg D. Melano T. McKinstry J. Di Nolfo C.. (2017). “A low power, fully event-based gesture recognition system,” in Proceedings of the IEEE Conference on Computer Vision and Pattern Recognition (Honolulu, HI), 7243–7252. 10.1109/CVPR.2017.781

[B6] Arthur J. V. Merolla P. A. Akopyan F. Alvarez R. Cassidy A. Chandra S.. (2012). “Building block of a programmable neuromorphic substrate: a digital neurosynaptic core,” in the 2012 International Joint Conference on Neural Networks (IJCNN) (Brisbane, QLD), 1–8. 10.1109/IJCNN.2012.6252637

[B7] Balaji A. Wu Y. Das A. Catthoor F. Schaafsma S. (2019). “Exploration of segmented bus as scalable global interconnect for neuromorphic computing,” in Proceedings of the 2019 on Great Lakes Symposium on VLSI (Tysons Corner, VA), 495–499. 10.1145/3299874.3319491

[B8] Bamberg L. Joseph J. M. García-Ortiz A. Pionteck T. (2022). “Interconnect architectures for 3d technologies,” in 3D Interconnect Architectures for Heterogeneous Technologies (Springer), 27–47. 10.1007/978-3-030-98229-4_2

[B9] Basu A. Deng L. Frenkel C. Zhang X. (2022). “Spiking neural network integrated circuits: a review of trends and future directions,” in 2022 IEEE Custom Integrated Circuits Conference (CICC) (Newport Beach, CA), 1–8. 10.1109/CICC53496.2022.9772783

[B10] Bellec G. Salaj D. Subramoney A. Legenstein R. Maass W. (2018). “Long short-term memory and learning-to-learn in networks of spiking neurons,” in Advances in Neural Information Processing Systems, Vol. 31 (Montreal).

[B11] Bellec G. Scherr F. Subramoney A. Hajek E. Salaj D. Legenstein R.. (2020). A solution to the learning dilemma for recurrent networks of spiking neurons. Nat. Commun. 11, 1–15. 10.1038/s41467-020-17236-y 32681001 PMC7367848

[B12] Benmeziane H. El Maghraoui K. Ouarnoughi H. Niar S. Wistuba M. Wang N. (2021). “Hardware-aware neural architecture search: survey and taxonomy,” in IJCAI, 4322–4329. 10.24963/ijcai.2021/592

[B13] Beyne E. Milojevic D. Van der Plas G. Beyer G. (2021). “3D SOC integration, beyond 2.5 d chiplets,” in 2021 IEEE International Electron Devices Meeting (IEDM) (San Francisco, CA), 3–6. 10.1109/IEDM19574.2021.9720614

[B14] Bohnstingl T. Woźniak S. Pantazi A. Eleftheriou E. (2022). Online spatio-temporal learning in deep neural networks. IEEE Trans. Neural Netw. Learn. Syst. 1–15. 10.1109/TNNLS.2022.3153985 35294357

[B15] Brown T. Mann B. Ryder N. Subbiah M. Kaplan J. D. Dhariwal P.. (2020). Language models are few-shot learners. Adv. Neural Inform. Process. Syst. 33, 1877–1901. Available online at: https://papers.nips.cc/paper_files/paper/2020/file/1457c0d6bfcb4967418bfb8ac142f64a-Paper.pdf35785085

[B16] Cadence (2021). Joules RTL power solution. Available online at: https://www.cadence.com/en_US/home/tools/digital-design-and-signoff/power-analysis/joules-rtl-power-solution.html

[B17] Chadwick Greg E. A. (2018). Ibex. Available online at: https://github.com/lowRISC/ibex

[B18] Chen L. Xiong X. Liu J. (2022). A survey of intelligent chip design research based on spiking neural networks. IEEE Access 10, 89663–89686. 10.1109/ACCESS.2022.3200454

[B19] Chitty-Venkata K. T. Somani A. K. (2022). Neural architecture search survey: a hardware perspective. ACM Comput. Surveys 55, 1–36. 10.1145/352450036772749

[B20] Coelho C. N. Kuusela A. Li S. Zhuang H. Ngadiuba J. Aarrestad T. K.. (2021). Automatic heterogeneous quantization of deep neural networks for low-latency inference on the edge for particle detectors. Nat. Mach. Intell. 3, 675–686. 10.1038/s42256-021-00356-5

[B21] Davies M. Srinivasa N. Lin T. Chinya G. Cao Y. Choday S. H.. (2018). Loihi: a neuromorphic manycore processor with on-chip learning. IEEE Micro 38, 82–99. 10.1109/MM.2018.112130359

[B22] Davis M. (2021). Taking neuromorphic computing to the next level with loihi 2. *Intel Technol. Brief* . Available online at: https://download.intel.com/newsroom/2021/new-technologies/neuromorphic-computing-loihi-2-brief.pdf

[B23] Demler M. (2019). Brainchip Akida is a Fast Learner, Spiking-Neural-Network Processor Identifies Patterns in Unlabeled Data. Microprocessor Report. Available online at: https://d1io3yog0oux5.cloudfront.net/brainchipinc/files/BrainChip+Akida+Is+a+Fast+Learner.pdf

[B24] Deng L. Wang G. Li G. Li S. Liang L. Zhu M.. (2020). Tianjic: a unified and scalable chip bridging spike-based and continuous neural computation. IEEE J. Solid State Circuits 55, 2228–2246. 10.1109/JSSC.2020.2970709

[B25] Flynn M. J. (1972). Some computer organizations and their effectiveness. IEEE Trans. Comput. 100, 948–960. 10.1109/TC.1972.5009071

[B26] Frenkel C. Indiveri G. (2022). “Reckon: a 28nm Sub-mm2 task-agnostic spiking recurrent neural network processor enabling on-chip learning over second-long timescales,” in 2022 IEEE International Solid-State Circuits Conference (ISSCC) (San Francisco, CA), *Vol. 65*, 1–3. 10.1109/ISSCC42614.2022.9731734

[B27] Frenkel C. Lefebvre M. Legat J.-D. Bol D. (2018). A 0.086-mm2 12.7-pj/sop 64k-synapse 256-neuron online-learning digital spiking neuromorphic processor in 28-nm cmos. IEEE Trans. Biomed. Circuits Syst. 13, 145–158. 10.1109/TBCAS.2018.2880425 30418919

[B28] Furber S. B. Galluppi F. Temple S. Plana L. A. (2014). The spinnaker project. Proc. IEEE 102, 652–665. 10.1109/JPROC.2014.2304638

[B29] Goetschalckx K. Wu F. Verhelst M. (2022). Depfin: a 12-nm depth-first, high-resolution CNN processor for IO-efficient inference. IEEE J. Solid-State Circuits. 58, 1425–1435. 10.1109/JSSC.2022.3210591

[B30] Grigorescu S. Trasnea B. Cocias T. Macesanu G. (2020). A survey of deep learning techniques for autonomous driving. J. Field Robot. 37, 362–386. 10.1002/rob.21918

[B31] Hartmann K. Thomson E. E. Zea I. Yun R. Mullen P. Canarick J.. (2016). Embedding a panoramic representation of infrared light in the adult rat somatosensory cortex through a sensory neuroprosthesis. J. Neurosci. 36, 2406–2424. 10.1523/JNEUROSCI.3285-15.2016 26911689 PMC4764662

[B32] Höppner S. Yan Y. Dixius A. Scholze S. Partzsch J. Stolba M.. (2021). The spinnaker 2 processing element architecture for hybrid digital neuromorphic computing. arXiv preprint arXiv:2103.08392.

[B33] Hwu T. Isbell J. Oros N. Krichmar J. (2017). “A self-driving robot using deep convolutional neural networks on neuromorphic hardware,” in 2017 International Joint Conference on Neural Networks (IJCNN) (Anchorage, AK), 635–641. 10.1109/IJCNN.2017.7965912

[B34] Jacob B. Kligys S. Chen B. Zhu M. Tang M. Howard A.. (2018). “Quantization and training of neural networks for efficient integer-arithmetic-only inference,” in Proceedings of the IEEE Conference on Computer Vision and Pattern Recognition (Salt Lake City, UT), 2704–2713. 10.1109/CVPR.2018.00286

[B35] Kalamkar D. Mudigere D. Mellempudi N. Das D. Banerjee K. Avancha S.. (2019). A study of bfloat16 for deep learning training. arXiv preprint arXiv:1905.12322.

[B36] Kheradpisheh S. R. Ganjtabesh M. Thorpe S. J. Masquelier T. (2018). STDP-based spiking deep convolutional neural networks for object recognition. Neural Netw. 99, 56–67. 10.1016/j.neunet.2017.12.005 29328958

[B37] Khoram S. Li J. (2018). “Adaptive quantization of neural networks,” in International Conference on Learning Representations (Vancouver, BC).

[B38] Kim M. Saad W. Mozaffari M. Debbah M. (2022). “On the tradeoff between energy, precision, and accuracy in federated quantized neural networks,” in ICC 2022-IEEE International Conference on Communications (Seoul), 2194–2199. IEEE. 10.1109/ICC45855.2022.9838362

[B39] Köster U. Webb T. Wang X. Nassar M. Bansal A. K. Constable W.. (2017). “Flexpoint: an adaptive numerical format for efficient training of deep neural networks,” in Advances in Neural Information Processing Systems, Vol. 30.

[B40] Kumar N. Tang G. Yoo R. Michmizos K. P. (2022). Decoding EEG with spiking neural networks on neuromorphic hardware. *Trans. Mach. Learn. Res*. Available online at: https://openreview.net/forum?id=ZPBJPGX3Bz

[B41] Kurtz M. Kopinsky J. Gelashvili R. Matveev A. Carr J. Goin M.. (2020). “Inducing and exploiting activation sparsity for fast neural network inference,” in Proceedings of the International Conference on Machine Learning.

[B42] LeCun Y. Bengio Y. Hinton G. (2015). Deep learning. Nature 521, 436–444. 10.1038/nature14539 26017442

[B43] LeDoux J. E. (1994). Emotion, memory and the brain. Sci. Am. 270, 50–57. 10.1038/scientificamerican0694-50 8023118

[B44] Lv C. Xu J. Zheng X. (2023). “Spiking convolutional neural networks for text classification,” in The Eleventh International Conference on Learning Representations (Kigali).

[B45] Lv M. Xu E. (2022). Efficient dnn execution on intermittently-powered iot devices with depth-first inference. IEEE Access 10, 101999–102008. 10.1109/ACCESS.2022.3203719

[B46] Massa R. Marchisio A. Martina M. Shafique M. (2020). “An efficient spiking neural network for recognizing gestures with a DVS camera on the Loihi neuromorphic processor,” in 2020 International Joint Conference on Neural Networks (IJCNN) (Glasgow, UK), 1–9. 10.1109/IJCNN48605.2020.9207109

[B47] Mayr C. Hoeppner S. Furber S. (2019). Spinnaker 2: A 10 million core processor system for brain simulation and machine learning. arXiv preprint arXiv:1911.02385.

[B48] Mink J. W. Blumenschine R. J. Adams D. B. (1981). Ratio of central nervous system to body metabolism in vertebrates: its constancy and functional basis. Am. J. Physiol. Regul. Integr. Compar. Physiol. 241, R203–R212. 10.1152/ajpregu.1981.241.3.R203 7282965

[B49] Molendijk M. Vadivel K. Corradi F. van Schaik G.-J. Yousefzadeh A. Corporaal H. (2022). “Benchmarking the epiphany processor as a reference neuromorphic architecture,” in Industrial Artificial Intelligence Technologies and Applications, 21–34.

[B50] Moons B. Goetschalckx K. Van Berckelaer N. Verhelst M. (2017). “Minimum energy quantized neural networks,” in 2017 51st Asilomar Conference on Signals, Systems, and Computers (Pacific Grove, CA), 1921–1925. 10.1109/ACSSC.2017.8335699

[B51] Moradi S. Qiao N. Stefanini F. Indiveri G. (2017). A scalable multicore architecture with heterogeneous memory structures for dynamic neuromorphic asynchronous processors (dynaps). IEEE Trans. Biomed. Circuits Syst. 12, 106–122. 10.1109/TBCAS.2017.2759700 29377800

[B52] Moreira O. Yousefzadeh A. Chersi F. Cinserin G. Zwartenkot R. J. Kapoor A.. (2020). “Neuronflow: a neuromorphic processor architecture for live AI applications,” in 2020 Design, Automation Test in Europe Conference Exhibition (DATE) (Grenoble), 840–845. 10.23919/DATE48585.2020.9116352

[B53] Negri P. Soto M. Linares-Barranco B. Serrano-Gotarredona T. (2018). “Scene context classification with event-driven spiking deep neural networks,” in 2018 25th IEEE International Conference on Electronics, Circuits and Systems (ICECS) (Bordeaux), 569–572. 10.1109/ICECS.2018.8617982

[B54] Patino-Saucedo A. Rostro-Gonzalez H. Serrano-Gotarredona T. Linares-Barranco B. (2020). Event-driven implementation of deep spiking convolutional neural networks for supervised classification using the spinnaker neuromorphic platform. Neural Netw. 121, 319–328. 10.1016/j.neunet.2019.09.008 31590013

[B55] Pedram A. Richardson S. Horowitz M. Galal S. Kvatinsky S. (2016). Dark memory and accelerator-rich system optimization in the dark silicon era. IEEE Des. Test 34, 39–50. 10.1109/MDAT.2016.2573586

[B56] Perrett A. Summerton S. Gait A. Rhodes O. (2022). “Online learning in snns with e-prop and neuromorphic hardware,” in Neuro-Inspired Computational Elements Conference, 32–39. 10.1145/3517343.3517352

[B57] Quian Quiroga R. Kreiman G. (2010). Measuring sparseness in the brain: comment on bowers (2009). Psychol. Review. 117, 291–297. 10.1037/a0016917 20063978 PMC3154835

[B58] Ravindran R. Santora M. J. Jamali M. M. (2020). Multi-object detection and tracking, based on dnn, for autonomous vehicles: a review. IEEE Sensors J. 21, 5668–5677. 10.1109/JSEN.2020.3041615

[B59] Renner A. Sheldon F. Zlotnik A. Tao L. Sornborger A. (2021). The backpropagation algorithm implemented on spiking neuromorphic hardware. arXiv preprint arXiv:2106.07030. 10.21203/rs.3.rs-701752/v1 PMC11549378 39516210

[B60] Rostami A. Vogginger B. Yan Y. Mayr C. G. (2022). E-prop on spinnaker 2: exploring online learning in spiking RNNs on neuromorphic hardware. Front. Neurosci. 16:6. 10.3389/fnins.2022.1018006 36518534 PMC9742366

[B61] Schemmel J. Billaudelle S. Dauer P. Weis J. (2022). “Accelerated analog neuromorphic computing,” in Analog Circuits for Machine Learning, Current/Voltage/Temperature Sensors, and High-speed Communication (Springer), 83–102. 10.1007/978-3-030-91741-8_6

[B62] Schiavone P. D. Conti F. Rossi D. Gautschi M. Pullini A. Flamand E.. (2017). “Slow and steady wins the race? A comparison of ultra-low-power RISC-V cores for internet-of-things applications,” in 2017 27th International Symposium on Power and Timing Modeling, Optimization and Simulation (PATMOS) (Thessaloniki), 1–8. 10.1109/PATMOS.2017.8106976

[B63] Shankar V. Roelofs R. Mania H. Fang A. Recht B. Schmidt L. (2020). “Evaluating machine accuracy on imagenet,” in International Conference on Machine Learning (Vienna), 8634–8644.

[B64] Sheikh F. Nagisetty R. Karnik T. Kehlet D. (2021). 2.5 d and 3d heterogeneous integration: emerging applications. IEEE Solid-State Circuits Mag. 13, 77–87. 10.1109/MSSC.2021.3111386

[B65] Silver D. Schrittwieser J. Simonyan K. Antonoglou I. Huang A. Guez A.. (2017). Mastering the game of go without human knowledge. Nature 550, 354–359. 10.1038/nature24270 29052630

[B66] Stansfield T. (2022). Improving the efficiency of AI applications using in-memory computation [White paper]. Surefcore Limited. Available online at: https://www.sure-core.com/new-wp/wp-content/uploads/2022/10/WP4-AI-IMC-1.pdf

[B67] Stromatias E. Galluppi F. Patterson C. Furber S. (2013). “Power analysis of large-scale, real-time neural networks on spinnaker,” in The 2013 International Joint Conference on Neural Networks (IJCNN) (Dallas, TX), 1–8. 10.1109/IJCNN.2013.6706927

[B68] Stuijt J. Sifalakis M. Yousefzadeh A. Corradi F. (2021). μbrain: an event-driven and fully synthesizable architecture for spiking neural networks. Front. Neurosci. 15:538. 10.3389/fnins.2021.664208 34093116 PMC8170091

[B69] Symons A. Mei L. Colleman S. Houshmand P. Karl S. Verhelst M. (2022). Towards heterogeneous multi-core accelerators exploiting fine-grained scheduling of layer-fused deep neural networks. arXiv preprint arXiv:2212.10612.

[B70] Tang G. Kumar N. Polykretis I. Michmizos K. P. (2021). Biograd: biologically plausible gradient-based learning for spiking neural networks. arXiv preprint arXiv:2110.14092.

[B71] Teman A. Rossi D. Meinerzhagen P. Benini L. Burg A. (2016). Power, area, and performance optimization of standard cell memory arrays through controlled placement. ACM Trans. Des. Autom. Electron. Syst. 21, 1–25. 10.1145/2890498

[B72] Tolstikhin I. O. Houlsby N. Kolesnikov A. Beyer L. Zhai X. Unterthiner T.. (2021). MLP-mixer: an all-MLP architecture for vision. Adv. Neural Inform. Process. Syst. 34, 24261–24272. Available online at: https://proceedings.neurips.cc/paper/2021/file/cba0a4ee5ccd02fda0fe3f9a3e7b89fe-Paper.pdf

[B73] Traub M. Otte S. Menge T. Karlbauer M. Thümmel J. Butz M. V. (2022). Learning what and where-unsupervised disentangling location and identity tracking. arXiv preprint arXiv:2205.13349.

[B74] Vaswani A. Shazeer N. Parmar N. Uszkoreit J. Jones L. Gomez A. N.. (2017). “Attention is all you need,” in Advances in Neural Information Processing Systems, Vol. 30 (Long Beach, CA).

[B75] Waterman A. Lee Y. Patterson D. Asanovic K. level Isa V. I. U. (2014). The RISC-v Instruction Set Manual. Vol. I: User-Level ISA, Version, 2. 10.21236/ADA605735

[B76] Wu Y. Deng L. Li G. Zhu J. Shi L. (2018). Spatio-temporal backpropagation for training high-performance spiking neural networks. Front. Neurosci. 12:331. 10.3389/fnins.2018.00331 29875621 PMC5974215

[B77] Xilinx (2020). Virtex ultrascale+ hbm fpga. Available online at: https://www.xilinx.com/products/silicon-devices/fpga/virtex-ultrascale-plus-hbm.html

[B78] Yin B. Corradi F. Bohté S. M. (2021). Accurate and efficient time-domain classification with adaptive spiking recurrent neural networks. Nat. Mach. Intell. 3, 905–913. 10.1038/s42256-021-00397-w

[B79] Yousefzadeh A. Jabłoński M. Iakymchuk T. Linares-Barranco A. Rosado A. Plana L. A.. (2017a). On multiple AER handshaking channels over high-speed bit-serial bidirectional LVDS links with flow-control and clock-correction on commercial FPGAS for scalable neuromorphic systems. IEEE Trans. Biomed. Circuits Syst. 11, 1133–1147. 10.1109/TBCAS.2017.2717341 28809708

[B80] Yousefzadeh A. Khoei M. A. Hosseini S. Holanda P. Leroux S. Moreira O.. (2019). Asynchronous spiking neurons, the natural key to exploit temporal sparsity. IEEE J. Emerg. Selec. Top. Circuits Syst. 9, 668–678. 10.1109/JETCAS.2019.2951121

[B81] Yousefzadeh A. Masquelier T. Serrano-Gotarredona T. Linares-Barranco B. (2017b). “Hardware implementation of convolutional stdp for on-line visual feature learning,” in 2017 IEEE International Symposium on Circuits and Systems (ISCAS) (Baltimore, MD), 1–4. 10.1109/ISCAS.2017.8050870

[B82] Yousefzadeh A. Plana L. A. Temple S. Serrano-Gotarredona T. Furber S. B. Linares-Barranco B. (2016). Fast predictive handshaking in synchronous FPGAS for fully asynchronous multisymbol chip links: application to spinnaker 2-of-7 links. IEEE Trans. Circuits Syst. II 63, 763–767. 10.1109/TCSII.2016.2531092

[B83] Yousefzadeh A. Serrano-Gotarredona T. Linares-Barranco B. (2015). “Fast pipeline 128 × 128 pixel spiking convolution core for event-driven vision processing in FPGAS,” in 2015 International Conference on Event-Based Control, Communication, and Signal Processing (EBCCSP), 1–8. IEEE. 10.1109/EBCCSP.2015.7300698

[B84] Yousefzadeh A. Sifalakis M. (2022). “Delta activation layer exploits temporal sparsity for efficient embedded video processing,” in 2022 International Joint Conference on Neural Networks (IJCNN) (Padua), 1–10. 10.1109/IJCNN55064.2022.9892578

[B85] Yousefzadeh A. Van Schaik G.-J. Tahghighi M. Detterer P. Traferro S. Hijdra M.. (2022). “Seneca: scalable energy-efficient neuromorphic computer architecture,” in 2022 IEEE 4th International Conference on Artificial Intelligence Circuits and Systems (AICAS) (Incheon), 371–374. 10.1109/AICAS54282.2022.9870025

[B86] Zambrano D. Nusselder R. Scholte H. S. Bohté S. M. (2019). Sparse computation in adaptive spiking neural networks. Front. Neurosci. 12:987. 10.3389/fnins.2018.00987 30670943 PMC6332470

